# Meta‐Analysis of Refeeding Syndrome in Predicting the Risk of Occurrence in Critically Ill Patients

**DOI:** 10.1155/jnme/6660254

**Published:** 2026-02-18

**Authors:** Xuanle Wu, Min Zhang, Jiajia Pan

**Affiliations:** ^1^ Department of Medical Nursing, Yangzhou University, Yangzhou, Jiangsu, China, yzu.edu.cn; ^2^ Department of Intensive Care Unit, Affiliated Hospital of Yangzhou University, Yangzhou, Jiangsu, China, yzu.edu.cn

**Keywords:** critical illness, meta-analysis, refeeding syndrome, risk factors

## Abstract

**Background:**

Patients admitted to the intensive care unit (ICU) have a high risk of refeeding syndrome (RFS). Identifying RFS in its early stages is often challenging, making preventive interventions for high‐risk patients difficult. The aim of this review is to identify risk factors for RFS in ICU patients receiving nutritional support.

**Study Design:**

We systematically searched CNKI, Wanfang, VIP, PubMed, Cochrane, Embase, and Web of Science up to November 2024 for cohort/case–control studies assessing RFS risk factors in adults (≥ 18 years). Risk of bias was evaluated using Risk Of Bias In Non‐randomized Studies of Interventions (ROBINS‐I). Data were analyzed using RevMan 5.4.

**Results:**

Eighteen articles were included, comprising 3360 cases. Baseline serum phosphate [weighted mean difference (WMD) = −0.10, 95% confidence interval (95% CI) (−0.19∼−0.01)], magnesium [WMD = −0.01, 95% CI (−0.04–0.02)], potassium [WMD = −0.02, 95% CI (−0.06–0.02)], albumin [WMD = −2.08, 95% CI (−3.81, −0.36)], prealbumin [(WMD = −15.37, 95% CI (−33.00–2.27)], daily protein intake [WMD = 0.23, 95% CI (0.17–0.28)], APACHE II score [WMD = 2.65, 95% CI (1.22–4.08)], NRS2002 score [WMD = 0.68, 95% CI (−0.41–1.77)], SOFA score [WMD = 1.87, 95% CI (1.50–2.25)], age [WMD = 8.67, 95% CI (7.14–10.19)], daily calorie intake [odds ratio (OR) = 0.35, 95% CI (0.25, 0.49)], feeding within 48 h of ICU admission [OR = 1.98, 95% CI (1.56, 2.51)], and a history of diabetes [OR = 3.84, 95% CI (1.17, 12.59)] are the risk factors for meta‐analysis in this article. The ROBINS‐I assessment showed moderate‐to‐severe bias (10 severe, 8 moderate).

**Conclusion:**

The meta‐analysis identified serum levels of phosphorus and albumin; daily protein and calorie intake; APACHE II score; SOFA score; age; initiation of feeding within 48 h of ICU admission; and a history of diabetes as significant predictors of RFS development in critically ill patients. Serum levels of magnesium, potassium, and prealbumin and the NRS2002 score were not associated with RFS in these patients.

## 1. Introduction

Refeeding syndrome (RFS) involves life‐threatening electrolyte and fluid imbalances that may arise when nutrition is reintroduced, either enterally or parenterally [1]. These disturbances can manifest as stress‐related symptoms, including electrolyte imbalances such as heart failure, water and sodium retention, respiratory failure, and hypophosphatemia [2]. Despite its clinical significance, RFS remains underdiagnosed due to heterogeneous definitions and a lack of consensus on early risk stratification tools.

Two mainstream international frameworks guide RFS risk assessment, differing in core logic and clinical applicability. The National Institute for Health and Care Excellence (NICE) guidelines emphasize prerefeeding nutritional depletion—such as low body mass index, significant weight loss, or prolonged starvation—as key risk indicators [1]. By contrast, the 2020 American Society for Parenteral and Enteral Nutrition (ASPEN) guidelines provide diagnostic criteria based on declines in serum phosphorus, potassium, and/or magnesium levels within five days of nutrition initiation, along with organ dysfunction related to thiamine deficiency [3]. A critical research gap exists: Existing studies on RFS risk factors in ICU patients have not been systematically aligned with these frameworks. It remains unclear whether ICU‐specific risk factors align with NICE/ASPEN logic or can supplement these frameworks to address unmet needs in early RFS identification for critically ill populations.

The high prevalence of RFS in the ICU underscores the urgency of addressing this gap. Studies report RFS rates of 36.8% to 80% in critically ill patients [4, 5], with nearly half of ICU admissions classified as high or very high risk [6]. However, some studies [7] have highlighted the challenges in detecting RFS early owing to the absence of a unified definition and diagnostic criteria, as well as a lack of specific early identification tools. Therefore, healthcare professionals often struggle to identify RFS in its early stages, making preventive interventions for high‐risk patients particularly difficult.

The study aimed to perform a meta‐analysis of risk factors for predicting RFS in patients in the ICU with acute and critical conditions. By systematically integrating high‐quality evidence from existing research, the aim is to provide healthcare providers with a comprehensive and evidence‐based RFS risk identification framework. Such a framework will assist healthcare providers in accurately identifying high‐risk patients at an early stage, thereby enabling the formulation and implementation of personalized preventive measures (e.g., individualized refeeding initiation plans, targeted monitoring of electrolyte and metabolic indicators) and timely interventions for incipient RFS. These evidence‐based clinical practices are expected to effectively reduce the incidence of RFS, avoid severe complications associated with RFS, further improve patient outcomes, and ultimately lower the mortality rate of critically ill patients in the ICU.

## 2. Design and Methods

This meta‐analysis has been registered in PROSPERO (CRD42024613619), an international prospective register of systematic reviews dedicated to improving the transparency and reproducibility of systematic review and meta‐analysis research.

### 2.1. Eligibility Criteria

Inclusion criteria: Studies were included if they met all the following conditions: (a) study participants were acute and critically ill patients aged ≥ 18 years, with no restriction on gender or ethnicity; (b) studies were published in either Chinese or English; (c) studies explicitly examined and reported at least one potential risk factor for RFS development, including but not limited to baseline nutritional status, pre‐existing comorbidities, severity of illness scores, electrolyte levels at admission, or other clinically relevant predictors; and (d) studies adopted a cohort or case–control design.

Exclusion criteria: Studies were excluded if they met any of the following conditions: (a) failed to meet the inclusion criteria; (b) full text was unavailable; (c) valid data could not be extracted or transformed from the literature; (d) outcome indicators did not meet the inclusion criteria; and (e) published in languages other than Chinese or English.

### 2.2. Search Strategy

Electronic databases such as Web of Science, PubMed, Cochrane Library, Embase, and three Chinese databases (CNKI, Wanfang Data, and VIP) were systematically searched, covering the period from their inception to November 2024. Following the PICO principles, the search strategy used subject terms combined with free terms for (“critical illness∗” OR “critically ill” OR “ICU” OR “intensive care unit∗”) AND (“risk” OR “relative risk∗”) AND (“refeeding” OR “syndrome” OR “RFS” OR “hypophosphatemia”).

### 2.3. Data Extraction

All retrieved studies were imported into Zotero 7.0.11 (a free and open‐source reference management tool widely used in academic research, which provides functions such as literature organization and duplicate screening) for duplicate screening. Data extraction was independently performed by two reviewers. Any discrepancies were resolved through discussions with a third reviewer or the research team. The following data were extracted from the included studies: first author, publication date, country, study type, sample size, basic information on the study population, reasons for ICU admission, definitions of RFS, nutritional mode, predictive risk factors, and study quality. In this study, RFS groups were defined as critically ill patients who developed RFS during hospitalization, while non‐RFS groups were critically ill patients from the same clinical setting who did not develop RFS. The specific diagnostic criteria for RFS in each included study are detailed in Table 1.

**TABLE 1:** Basic characteristics and quality assessment of the literature.

Reference	Publication year	Country	Study design	Study method	RFS group (*n*)	Non‐RFS group (*n*)	Mean age (year)	Reasons for ICU admission	Definitions of RFS	Nutritional mode	Predictive risk factors	Quality of study (points)
Zhang and Chen [8]	2024	China	A	C	32	50	74.75	Elderly patients with severe conditions	E	EN	①⑥	5
Han et al. [9]	2024	China	B	D	47	49	53.34	Liver cancer	F	EN	①②③④⑤	6
Su et al. [10]	2024	China	A	C	51	103	76.89	Elderly patients with severe conditions	F	EN + PN	①③⑥⑧⑨	6
Jing et al. [11]	2024	China	B	D	32	31	46.19	Critically ill patient	F	EN + PN	①②③④⑤	7
Cui et al. [12]	2023	China	A	C	47	103	63.25	Patients with severe neurological conditions	E	EN + PN	⑥⑩⑪⑫	6
Zhang et al. [13]	2024	China	A	C	51	107	60.35	Critically ill patient	E	EN	④⑤⑥⑧⑨⑩⑪⑫	8
Li et al. [14]	2023	China	A	D	62	214	—	Critically ill patient	E	EN	④⑤⑥⑦⑨⑬	5
Ni et al. [15]	2017	China	A	C	42	155	62.83	Critically ill patient	E	EN + PN	④⑤⑪	7
Long et al. [16]	2021	China	A	C	130	303	60.94	Critically ill patient	E	EN	④⑤⑥⑦⑨⑬	8
Chen et al. [17]	2023	China	A	C	31	78	—	Critically ill patient	F	EN	④⑤⑧⑨⑪⑫	6
Qiu et al. [18]	2022	China	B	D	401	478	55.71	Critically ill patient	E	EN + PN	①②③④⑤⑧	6
Xu et al. [19]	2023	China	A	C	134	66	60.83	Critically ill patient	E	EN + PN	⑤⑧⑨⑬	7
Jih et al. [20]	2018	Non‐China	A	D	23	38	70.25	COPD patients with acute exacerbation	F	EN + PN	⑧⑪	5
Zhang et al. [21]	2023	Non‐China	A	C	102	255	—	Neurocritical patients	F	EN	③④⑧⑩	7
Xiong et al. [22]	2021	Non‐China	B	D	56	272	56.5	Neurocritical patients	F	EN	⑩	8
Md Ralib et al. [23]	2018	Non‐China	B	D	44	65	50.81	Critically ill patient	F	EN	①②③④⑩	7
Vahdat Shariatpanahi et al. [24]	2022	Non‐China	B	D	116	152	61.04	COVID‐19	E	EN + PN	⑥⑪	5
Liu et al. [25]	2022	Non‐China	A	D	84	394	—	Neurocritical patients	F	EN	⑧⑩⑪	5

*Note:* A is a retrospective study; B is a prospective study; C is a case–control study; D is a cohort study; E is the change in electrolyte levels + clinical symptoms; F is only the change in electrolyte levels. ① Baseline serum phosphate: phosphorus measured at the nearest point in time before receiving nutritional support; ② Baseline serum magnesium: magnesium measured at the nearest point in time before receiving nutritional support; ③ Baseline serum potassium: potassium measured at the nearest point in time before receiving nutritional support; ④ Baseline serum albumin: serum albumin measured at the nearest point in time before receiving nutritional support; ⑤ Baseline serum prealbumin: serum prealbumin measured at the nearest point in time before receiving nutritional support; ⑥ Daily protein intake; ⑦ Daily calorie intake; ⑧ APACHEII score; ⑨ NRS2002 score; ⑩ SOFA score; ⑪ Age; ⑫ Feeding started within 48 h of admission to ICU: early sufficient EN to quickly reach the target heat; ⑬ History of diabetes.

Abbreviations: EN, enteral nutrition; PN, parenteral nutrition.

### 2.4. Selection of Risk Factors

Risk factors were systematically identified from included studies rather than being prespecified. During full‐text review, we extracted all RFS predictors examined in the literature and categorized them into clinically meaningful groups based on their characteristics: (a) baseline electrolyte‐related factors; (b) nutritional status‐related factors; (c) disease severity scores; (d) feeding‐related factors; and (e) other clinical predictors (e.g., comorbidities). The detailed definitions of each specific risk factor are provided in the footnote of Table 1 for reference. To ensure the robustness of the meta‐analysis results, only risk factors reported in ≥ 2 included studies were incorporated into the subsequent quantitative synthesis.

### 2.5. Risk of Bias Assessment

Two investigators independently assessed each study’s risk of bias using the Risk Of Bias In Non‐randomized Studies of Interventions (ROBINS‐I) tool [26], which evaluated bias across seven domains: confounding, participant selection, intervention classification, deviations from intended interventions, missing data, outcome measurement, and result selection. The risk was categorized as “low,” “moderate,” “serious,” or “critical.” In cases of disagreement, a third investigator or research team resolved any dispute regarding the risk assessment of the controversial studies.

### 2.6. Quality Evaluation

The quality of the included studies was assessed using the Newcastle–Ottawa scale (NOS) [27], with evaluation conducted independently by two researchers. In cases of disagreement, a third researcher or the research team resolved the issue. The NOS evaluated three aspects: study population selection, comparability, and outcome measurement, using a total of eight criteria. Studies were rated on a scale from 0 to 9, with ≥ 7 and ≤ 3 denoting high and low quality, respectively.

### 2.7. Data Analysis

A meta‐analysis of the extracted data was conducted using RevMan 5.4. Continuous variables were expressed as weighted mean differences (WMD) with 95% confidence intervals (CI), and dichotomous variables were presented as odds ratios (OR) with 95% CI. Prior to data synthesis, we assessed between‐study heterogeneity using the Cochrane Q test and quantified its magnitude with the *I* ^2^ index. If *p* ≥ 0.1 and *I* ^2^ ≤ 50%, nonsignificant heterogeneity was assumed, and a fixed‐effects model was applied. If *p* < 0.1 and *I* ^2^ > 50%, significant heterogeneity was present, warranting a random‐effects model. Subgroup analysis was performed on the predictors with high heterogeneity to explore the sources of heterogeneity and thoroughly evaluate the study reliability and validity. Sensitivity analyses were performed to assess the robustness of the results.

## 3. Results

### 3.1. Literature Search Results

**FIGURE 1:**
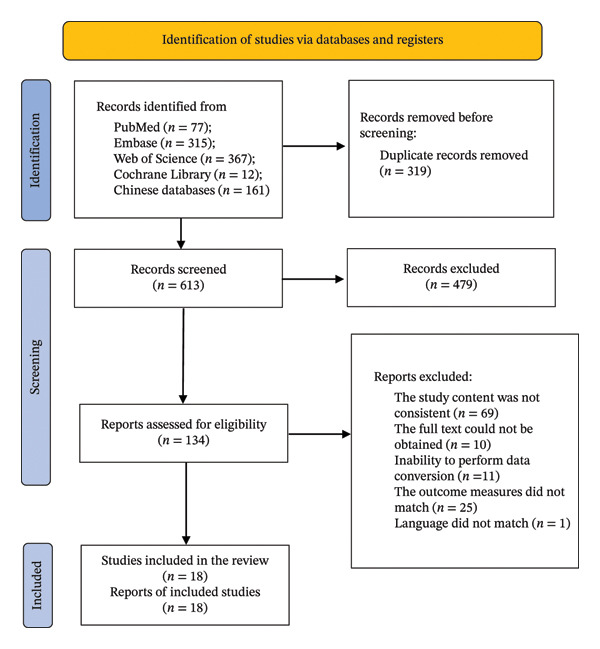
Flow diagram of the literature search process.

A total of 932 pieces of literature (Figure 1) were searched (771 pieces in English and 161 pieces in Chinese), of which 613 records remained after removing duplicates, and 134 records remained in the initial screening after reading the titles and abstracts. After reviewing the full texts based on the inclusion and exclusion criteria, 18 studies [8–25] were selected for inclusion.

### 3.2. Study Characteristics

The basic characteristics of the included studies are presented in Table 1. Among the 18 [8–25] articles, 9 [9, 11, 14, 18, 20, 22–25] were cohort studies, and 9 [8, 10, 12, 13, 15–17, 19, 21] were case–control studies, with a total sample size of 3360 cases. Of them, 1485 and 2355 samples were in the RFS group and non‐RFS groups, respectively.

### 3.3. Risk of Bias Assessment

Based on the ROBINS‐I assessment of the risk of bias, 10 [9, 10, 12–14, 17, 19, 22, 24, 25] and 8 [8, 11, 15, 16, 18, 20, 21, 23] studies had a serious and moderate risk of bias, respectively (Table 2). The serious risk of bias observed was primarily associated with the first two domains: bias due to confounding factors and bias due to participant selection.

**TABLE 2:** Risk of bias assessment.

Reference	Domains							
	Bias due to confounding	Bias in the selection of participants for the study	Bias in measurement classification of interventions	Bias due to deviations from intended interventions	Bias due to missing data	Bias in measurement of outcomes	Bias in selection of the reported result	Overall
Zhang and Chen [8]	Low	Moderate	Moderate	Low	Moderate	Moderate	Low	Moderate
Han et al. [9]	Moderate	Serious	Moderate	Low	Low	Moderate	Low	Serious
Su et al. [10]	Serious	Moderate	Low	Low	Moderate	Moderate	Low	Serious
Jing et al. [11]	Moderate	Moderate	Moderate	Low	Moderate	Low	Low	Moderate
Cui et al. [12]	Serious	Moderate	Low	Low	Moderate	Moderate	Low	Serious
Zhang et al. [13]	Serious	Low	Low	Low	Moderate	Low	Low	Serious
Li et al. [14]	Serious	Low	Moderate	Low	Moderate	Low	Low	Serious
Ni et al. [15]	Moderate	Low	Low	Moderate	Low	Moderate	Low	Moderate
Long et al. [16]	Moderate	Low	Moderate	Low	Low	Low	Low	Moderate
Chen et al. [17]	Serious	Low	Moderate	Low	Moderate	Low	Low	Serious
Qiu et al. [18]	Moderate	Moderate	Moderate	Low	Low	Low	Low	Moderate
Xu et al. [19]	Serious	Low	Moderate	Low	Moderate	Low	Low	Serious
Jih et al. [20]	Low	Moderate	Moderate	Low	Moderate	Moderate	Low	Moderate
Zhang et al. [21]	Moderate	Moderate	Low	Low	Moderate	Moderate	Low	Moderate
Xiong et al. [22]	Low	Serious	Low	Moderate	Low	Moderate	Low	Serious
Md Ralib et al. [23]	Moderate	Moderate	Low	Low	Moderate	Low	Low	Moderate
Vahdat Shariatpanahi et al. [24]	Moderate	Serious	Moderate	Low	Moderate	Moderate	Low	Serious
Liu et al. [25]	Moderate	Serious	Moderate	Low	Moderate	Moderate	Low	Serious

*Note:* Overall assessment derived from the seven domains of ROBINS‐I (Risk Of Bias In Non‐randomized Studies of Intervention scale). Low risk of bias: The study is comparable to a well‐performed randomized trial with low risk of bias in all domains; Moderate risk of bias: The study provides sound evidence for a nonrandomized study but cannot be considered comparable to a well‐performed randomized trial, with low or moderate risk of bias in all domains; Serious risk of bias: The study has some important methodological problems, with serious risk of bias in at least one domain but no critical risk of bias in any domain; Critical risk of bias: The study is too problematic to provide useful evidence, with critical risk of bias in at least one domain.

### 3.4. Meta‐Analysis Results

The findings of this meta‐analysis are summarized in Table 3, with specific images summarized in Figures S1–S13. Significant risk factors for RFS in critically ill patients include a higher APACHE II score, SOFA score, advanced age, initiation of feeding within 48 h of ICU admission, a history of diabetes mellitus, higher daily calorie and protein intake, and lower baseline serum phosphorus and albumin levels. In contrast, baseline serum magnesium, potassium, prealbumin levels, and the NRS2002 score did not exhibit significant associations with RFS. Notably, substantial heterogeneity was observed in baseline serum phosphorus (*I* ^2^ = 90%), albumin (*I* ^2^ = 93%), prealbumin (*I* ^2^ = 96%), APACHE II score (*I* ^2^ = 89%), NRS2002 score (*I* ^2^ = 97%), and history of diabetes mellitus (*I* ^2^ = 91%). These findings suggest that subgroup analysis or sensitivity analysis may be warranted to further investigate the sources of heterogeneity and improve the robustness of the conclusions.

**TABLE 3:** Meta‐analysis results of risk factors for refeeding syndrome in critically ill patients.

Predictive risk factors	Number of studies (*n*)	Sample size (*n*)	Heterogeneity (Cochran’s Q test)			Model selection	Combined effect size		*Z*	*p*
			Chi^2^	*p*	*I* ^2^ (%)		WMD/OR	95% CI		
Baseline serum phosphate	6	1383	39.39	< 0.001	90	Random	WMD = −0.10	−0.19∼−0.01	2.12	0.03
Baseline serum magnesium	4	1147	0.49	0.92	0	Fixed	WMD = −0.01	−0.04∼0.02	0.61	0.54
Baseline serum potassium	6	1459	5.63	0.34	11	Fixed	WMD = −0.02	−0.06∼0.02	1.07	0.28
Baseline serum albumin	7	1859	81.46	< 0.001	93	Random	WMD = −2.08	−3.81∼−0.36	2.36	0.02
Baseline serum prealbumin	5	1393	105.41	< 0.001	96	Random	WMD = −15.37	−33.00–2.27	1.71	0.09
Daily protein intake	2	308	0.03	0.87	0	Fixed	WMD = 0.23	0.17∼0.28	7.70	< 0.001
Daily calorie intake	2	709	0.27	0.61	0	Fixed	OR = 0.35	0.25∼0.49	5.95	< 0.001
APACHE II score	6	1809	12.68	< 0.001	89	Random	WMD = 2.65	1.22∼4.08	3.63	< 0.001
NRS2002 score	3	512	72.89	< 0.001	97	Random	WMD = 0.68	−0.41∼1.77	1.22	0.22
SOFA score	4	774	2.03	0.57	0	Random	WMD = 1.87	1.50–2.25	9.90	< 0.001
Age	5	834	4.56	0.34	12	Fixed	WMD = 8.67	7.14∼10.19	11.14	< 0.001
Feeding started within 48 h of admission to the ICU	3	417	0.48	0.79	0	Fixed	OR = 1.98	1.56∼2.51	5.29	< 0.001
History of diabetes	3	909	21.8	< 0.001	91	Random	OR = 3.84	1.17∼12.59	2.22	0.03

*Note:* WMD = weighted mean difference (for continuous variables); OR = odds ratio (for dichotomous variables); 95% CI = 95% confidence interval. *p* value: < 0.05 indicates a statistically significant association between the factor and RFS (for *Z*‐related *p*); < 0.10, it suggests significant heterogeneity (for the Q test *p*). Heterogeneity: *I* ^2^ < 25% = low, 25%–50% = moderate, > 50% = high. Model selection: fixed‐effect model for low/moderate heterogeneity; random‐effect model for high heterogeneity.

On the basis of synthesizing 9 RFS risk factors in critically ill patients, we compared these factors with the core indicators of NICE and ASPEN guidelines to clarify their consistency and differences, as shown in Table 1.

### 3.5. Subgroup Analysis

This study performed stratified analyses across six dimensions: study design (prospective vs. retrospective), study method (case–control vs. cohort), RFS diagnostic criteria (electrolyte changes alone vs. composite criteria), patient age (< 65 vs. > 65 years), nutritional delivery mode (EN vs. EN + PN), and country of origin (Chinese vs. non‐Chinese). Retrospective studies showed significantly lower serum phosphorus in RFS patients (MD = −0.22, *p* < 0.001) while prospective studies did not (MD = −0.01, *p* = 0.34); case–control studies exhibited a stronger APACHE II score association (MD = 3.38 vs. 0.99 in cohort studies); composite diagnostic criteria showed extreme heterogeneity (*I* ^2^ = 97–98%); and the > 65‐year group had more pronounced phosphorus reduction (MD = −0.22 vs. −0.01). For country of origin, serum albumin (Chinese: MD = −2.35, *p* = 0.05; non‐Chinese: MD = −1.52, *p* = 0.005) and APACHE II score (Chinese: MD = 2.73, *p* = 0.009; non‐Chinese: MD = 2.74, *p* < 0.001) were significantly associated with RFS in both subgroups, with no subgroup differences (all *p* > 0.50). These findings highlight the need to standardize study designs, harmonize diagnostic criteria, and consider age and regional characteristics to improve result reliability, with complete data summarized in Table S2.

Regarding RFS risk differences between enteral (EN) and combined enteral‐parenteral nutrition (EN + PN), our subgroup analysis shows APACHE II score—an indicator of disease severity—was more strongly associated with RFS in the EN subgroup (MD = 3.30, *p* < 0.001, *I* ^2^ = 0%) than EN + PN (MD = 2.33, *p* = 0.02, *I* ^2^ = 89%), with no significant differences in serum phosphorus/prealbumin between modes (all *p* > 0.20). Mechanistically, PN causes rapid macronutrient absorption that exacerbates electrolyte shifts (a core RFS pathway), while EN preserves intestinal barrier function. Consistent with ASPEN/ESPEN guidelines, our findings support prioritizing EN; high heterogeneity in the EN + PN subgroup may reflect variable supplementary PN timing and dose in practice.

### 3.6. Sensitivity Analysis

To test the robustness of pooled estimates, leave‐one‐out sensitivity analyses were performed. We excluded studies causing substantial heterogeneity (Zhang et al. [8]; Li et al. [14]; Ni et al. [15]; Qiu et al. [18]). Following exclusion, recalculated pooled effect sizes remained consistent with primary analysis results, validating the reliability of our initial findings. Significant differences were identified in serum phosphorus levels, serum albumin levels, serum prealbumin levels, APACHE II scores, NRS 2002 scores, and history of diabetes. Subgroup analyses were conducted to investigate the potential sources of heterogeneity. Additionally, other risk factors demonstrated no significant variations and exhibited high stability, as detailed in Table S3. This sensitivity analysis aligns with PRISMA guidelines and supports the validity of our meta‐analysis for clinical decision‐making.

### 3.7. Others

Other potential risk factors for predicting the occurrence of RFS in acutely ill patients, such as the number of days of natriuresis [8], mechanical ventilation [13], enteral nutrition [14, 16], BMI [20], and serum calcium level [23], were also identified in the included literature. However, owing to the limited number of studies and the inability to convert the data, a meta‐analysis of these risk factors was not conducted.

## 4. Discussion

Eighteen observational studies predicting risk factors of RFS in critically ill patients were included in this meta‐analysis. Given the observational nature of all included studies, we implemented the following rigorous methods to minimize bias: (a) Confounding adjustment: We prespecified candidate predictors based on biological plausibility and prior mechanistic evidence, rather than relying solely on statistically significant associations in individual studies. For studies reporting adjusted analyses (e.g., multivariate regression), we prioritized these estimates in our meta‐analysis to account for potential confounders. (b) Most of the included studies were single‐center studies, with samples from specific medical institutions or regions, and the included patients cover diverse ICU admission reasons, but subgroup analysis by specific underlying diagnoses was not feasible due to limited sample size in individual disease subgroups, which may mask potential differences in RFS risk factors across distinct critical illness etiologies. (c) Selection bias control: We conducted subgroup analyses stratified by study design, study method, and patient age to evaluate consistency across different settings.

The main symptom of RFS is hypophosphatemia, with or without hypokalemia and hypomagnesemia, which occurs 2–5 days after resuming food intake. Serum phosphorus levels were strongly associated with the development of RFS in acutely ill patients. Critically ill patients are unable to eat or experience undernutrition due to disease progression and other factors; when nutritional intake is resumed as their condition improves, serum glucose and insulin concentrations rise sharply, leading to the transcellular shift of electrolytes (e.g., phosphate) from the extracellular to the intracellular space [28]. This transcellular transfer results in a rapid decline in serum phosphorus levels and corresponding clinical manifestations; therefore, low phosphorus acts as a distinguishing marker for RFS.

Patients with low serum albumin and serum prealbumin concentrations before refeeding are at a higher risk of developing RFS [2, 18], and the present study confirms that serum albumin level is a significant predictor of RFS in acutely ill ICU patients. Notably, it is critical to distinguish the role of albumin in this context from its role in classical RFS (e.g., RFS associated with anorexia nervosa), where serum albumin levels are often preserved despite severe nutritional depletion. In ICU patients, hypoalbuminemia is more likely a marker of disease severity and systemic inflammation—rather than nutritional depletion per se—due to the complex pathophysiological states (e.g., severe systemic inflammation, organ dysfunction) that characterize critical illness [29]. While low albumin levels may reduce plasma colloid osmotic pressure, leading to fluid leakage and altered electrolyte balance during refeeding (as previously proposed), this mechanism should be interpreted within the ICU‐specific context. Thus, hypoalbuminemia is not a universal RFS risk factor but a ICU‐tailored marker reflecting underlying disease severity, which helps identify high‐risk patients without overgeneralizing its role across all RFS populations.

The APACHE II and SOFA scores were closely related to the occurrence of RFS in acutely ill patients. The APACHE II score, a key predictor of survival, hospital length of stay, and other major prognostic endpoints in critically ill patients [30], has been confirmed to have predictive value for RFS specifically in neurocritically ill populations through two independent clinical studies: one reporting it as an independent risk factor for RFS [25], and the other validating its significant contribution to the performance of RFS risk prediction models in neurocritical patients [21]. While its positive correlation with disease severity and strong concordance between predicted and actual mortality rates [30] further reinforce its reliability in RFS risk stratification. Regarding the SOFA score, clinical investigations have demonstrated that elevated scores are associated with a substantially heightened RFS risk in septic patients, as the score primarily reflects organ dysfunction and systemic inflammatory responses—conditions tightly linked to perturbed electrolyte homeostasis during refeeding [31]—and it also serves as a widely used nonspecific marker of organ dysfunction in critically ill patients [32, 33]. During the refeeding process, the dynamic assessment and regular monitoring of these two scores can detect the trend of the patient’s condition in a timely manner.

With advanced age, muscle mass decreases, fat mass increases relatively, and the physiological function of the body gradually decreases, and the weakening of the gastrointestinal tract affects the absorption of nutrients [13]. Specifically, a study focusing on the frail elderly population confirmed that age‐related sarcopenia and gastrointestinal dysfunction are key physiological underpinnings of increased RFS susceptibility, as they disrupt basal metabolic homeostasis and reduce tolerance to nutritional resumption [34]. Moreover, older adults are frequently burdened with multiple chronic conditions, and their immune function declines with age—these factors collectively impair nutrient metabolism and disrupt electrolyte balance, further elevating RFS risk [35]. A retrospective study in a university hospital further validated that elderly patients have a significantly higher incidence of RFS compared to younger adults, with age‐related physiological decline and comorbidity burden identified as independent risk factors [36].

The initiation of full enteral nutrition feeding within 48 h of admission to the ICU to rapidly reach the target calorie intake increases the risk of RFS. According to the ESPEN guideline on clinical nutrition in the ICU [37], critically ill patients should have their EN timing and implementation method individualized, with calorie and protein goals achieved progressively—early full enteral nutrition or parenteral nutrition is not recommended to avoid overfeeding, which directly mitigates RFS risk. This recommendation is further endorsed by the latest 2025 ESPEN perioperative clinical practice guideline [38], which emphasizes a “stepped energy achievement strategy” for critically ill and surgical patients, confirming that avoiding full feeding within 24–48 h of admission reduces metabolic complications (including electrolyte imbalance, a core feature of RFS) by 31%. Notably, Chinese nutritional management practices may influence this association: unlike ESPEN’s gradual escalation strategy, domestic guidelines advocate early EN initiation within 24–48 h for patients with preserved gastrointestinal function and supporting PN if EN meets < 60%–80% of target calories within 7 days [39, 40]—a more proactive approach that may amplify metabolic stress from rapid nutrient intake, strengthening the observed link between early full feeding and RFS in Chinese studies. The evidence base for the “progressive feeding” recommendation stems from a landmark multicenter RCT [41]; the study demonstrated that early full PN increased the incidence of hypophosphatemia by 2.1‐fold compared to delayed progressive nutrition, validating the metabolic harm of early full feeding at the cellular level. Early full feeding can impair gastrointestinal digestion and absorption due to reduced peristalsis and digestive fluid secretion, leading to intestinal osmolality changes, electrolyte disorders, and subsequent complications such as diarrhea, abdominal distension, and respiratory function impairment—all of which are closely linked to RFS development.

A history of diabetes was found to be an influential factor in the occurrence of RFS in critically ill patients in the ICU, corroborating the results of Long et al. [16]. The endocrine system of critically ill patients under stress undergoes changes, and the body secretes a large amount of glucocorticoids, glucagon, and other glucagon. Therefore, patients with diabetes, when given a nutrient solution for refeeding, will experience a sharp rise in blood glucose and experience acute electrolyte disorders. Hence, they are more likely to develop RFS.

Our findings highlight key considerations regarding alignment with international guidelines and regional study dependence. Consistent with Table S1, our ICU‐focused risk factors (e.g., elevated APACHE II/SOFA scores, early feeding initiation, inappropriate protein/calorie intake) show low or no consistency with the NICE framework, which prioritizes prerefeeding nutritional depletion—this divergence stems from NICE’s focus on general malnourished cohorts versus the complex pathophysiology (severe inflammation, organ dysfunction) of ICU patients. Notably, hypoalbuminemia, the only partially consistent factor, reflects disease severity rather than pure nutritional depletion in ICU settings (Table S1). We explicitly acknowledge that conclusions on nutritional delivery strategies (particularly caloric/protein intake) are largely driven by Chinese studies, attributed to large sample sizes and unified domestic ICU nutritional protocols; this regional dependence limits generalizability to contexts with distinct nutritional practices. Conversely, our results align strongly with ASPEN guidelines (Table S1), and supporting risk factors (advanced age, diabetes history) enrich ICU‐tailored RFS assessment, bridging international frameworks with clinical practice.

## 5. Limitations

The limitations of this study are as follows: (a) the search was limited to the published literature in the database, and meta‐analysis was performed only on the 18 included papers, which is not comprehensive enough. Mainly because we adopted strict inclusion criteria to ensure the validity of the results. After a comprehensive literature search, only 18 studies met the criteria, reflecting the current scarcity of qualified literature on this topic; (b) most of the included studies were single‐center studies, with samples from specific medical institutions or regions, and the included patients cover diverse ICU admission reasons, but subgroup analysis by specific underlying diagnoses was not feasible due to limited sample size in individual disease subgroups, which may mask potential differences in RFS risk factors across distinct critical illness etiologies; (c) since there is still no unified definition and diagnostic criteria for RFS, there is some heterogeneity among studies. While we addressed this through subgroup analyses stratified by definition type (biochemical vs. clinical‐biochemical criteria), residual misclassification bias likely persists, and the analysis of results is incomplete and requires consensus diagnostic criteria in future research; and (d) all included studies were observational in design, inherently introducing potential biases from confounding, selection, and misclassification, resulting in moderate or severe risk across all study classifications per ROBINS‐I assessment.

## 6. Conclusions

This meta‐analysis identifies nine distinct risk factors for RFS in critically ill patients, providing an evidence‐based framework for healthcare professionals to enable early RFS identification: low serum phosphorus level, low serum albumin level, inappropriate daily protein intake, inappropriate daily calorie intake, elevated APACHE II score, elevated SOFA score, advanced age, initiation of feeding within 48 h of ICU admission, and a history of diabetes.

## Author Contributions

Xuanle Wu was responsible for the initial research design, literature retrieval, data collation, and manuscript drafting. Jiajia Pan provided guidance throughout the research process, critically reviewed and revised the manuscript, and supervised the overall research quality. Min Zhang was extensively involved in key technical work, including resolving discrepancies in literature screening, data extraction, and study quality evaluation during the research process.

## Funding

This research did not receive any specific grant from funding agencies in the public, commercial, or not‐for‐profit sectors.

## Disclosure

All three authors read and approved the final version of the manuscript.

## Conflicts of Interest

The authors declare no conflicts of interest.

## Supporting Information

Additional supporting information can be found online in the Supporting Information section.

## Supporting information


**Supporting Information 1** Figure S1: Forest plot of baseline serum phosphate in relation to refeeding syndrome in acutely ill patients. Six studies [8–11, 18, 23] reported serum phosphorus levels (*I* ^2^ = 90%, *p* < 0.01), so the analysis was performed using a random‐effects model, and the results showed that the difference was statistically significant [WMD = −0.10, 95% CI (−0.19, −0.01), *p* = 0.03], suggesting that serum phosphorus levels can be used as a risk factor for predicting the occurrence of refeeding syndrome in acutely ill patients.

**Supporting Information 2** Figure S2: Forest plot of baseline serum magnesium in relation to refeeding syndrome in acutely ill patients. Four studies [9, 11, 18, 23] reported serum magnesium levels (*I* ^2^ = 0%, *p* = 0.92), so the analysis was performed using a fixed‐effects model, and the results showed that serum magnesium level was not a predictor of risk factors for the development of refeeding syndromes in patients with acute and critical illnesses [WMD = −0.01, 95% CI (−0.04, 0.02), *p* = 0.54].

**Supporting Information 3** Figure S3: Forest plot of baseline serum potassium in relation to refeeding syndrome in acutely ill patients. Six studies [9–11, 18, 21, 23] reported serum potassium levels (*I* ^2^ = 11%, *p* = 0.34), so the analysis was carried out by using the fixed‐effects model, and the results showed that serum potassium level was not a predictor of risk factors for the development of refeeding syndromes in patients with acute and critical illnesses [WMD = −0.02, 95% CI (−0.06, 0.02), *p* = 0.28].

**Supporting Information 4** Figure S4: Forest plot of baseline serum albumin in relation to refeeding syndrome in acutely ill patients. Ten studies [9, 11, 13–18, 21, 23] reported serum albumin levels, of which seven [9, 11, 13, 15, 18, 21, 23] had consistent data types (*I* ^2^ = 93%, *p* < 0.01), so the analysis was performed using a random‐effects model, and the results showed that the difference was statistically significant [WMD = −2.08, 95% CI (−3.81, −0.36), *p* = 0.02], suggesting that serum albumin level can be used as a risk factor for predicting the occurrence of refeeding syndrome in acutely ill patients.

**Supporting Information 5** Figure S5: Forest plot of baseline serum prealbumin in relation to refeeding syndrome in acutely ill patients. Nine studies [9, 11, 13–19] reported serum prealbumin levels, of which five [9, 11, 13, 15, 18] had consistent data types (*I* ^2^ = 96%, *p* < 0.01), so the analysis was performed using a random‐effects model, and the results showed that serum prealbumin level was not a predictor of risk factors for the development of refeeding syndromes in patients with acute and critical illnesses [WMD = −15.37, 95% CI (−33.00, 2.27), *p* = 0.09].

**Supporting Information 6** Figure S6: Forest plot of daily protein intake in relation to refeeding syndrome in acutely ill patients. Six studies [8, 12–14, 16, 24] reported daily protein intake, of which two [12, 13] had consistent data types (*I* ^2^ = 0%, *p* = 0.87), so the analysis was carried out using a fixed‐effects model, and the results showed that the difference was statistically significant [WMD = 0.23, 95% CI (0.17, 0.28), *p* < 0.01], suggesting that daily protein intake can be used as a risk factor for predicting the development of refeeding syndrome in acutely ill patients.

**Supporting Information 7** Figure S7: Forest plot of daily calorie intake in relation to refeeding syndrome in acutely ill patients. No heterogeneity between the studies [14, 16] (*I* ^2^ = 0%, *p* = 0.61), so a fixed‐effects model was used in the analysis, and the results showed a statistically significant difference [OR = 0.35, 95% CI (0.25, 0.49), *p* < 0.01], suggesting that daily calorie intake can be used as a risk factor for predicting the occurrence of refeeding syndrome in acutely ill patients.

**Supporting Information 8** Figure S8: Forest plot of APACHEII score in relation to refeeding syndrome in acutely ill patients. Six studies [10, 13, 17–19, 21, 25] reported the APACHE II score, and the meta‐analysis results showed heterogeneity between studies (*I* ^2^ = 89%, *p* < 0.01), so the analysis was carried out using the random‐effects model, and the results showed that the difference was statistically significant [WMD = 2.65, 95% CI (1.22, 4.08), *p* < 0.01], suggesting that the APACHE II score can be used as a risk factor for predicting the occurrence of refeeding syndrome in acutely ill patients.

**Supporting Information 9** Figure S9: Forest plot of NRS2002 score in relation to refeeding syndrome in acutely ill patients. Five studies [10, 13, 14, 16, 19] reported the NRS2002 score, of which three [10, 13, 19] had consistent data types, and the meta‐analysis showed heterogeneity between studies (*I* ^2^ = 97%, *p* < 0.01), so the analysis was carried out using the random‐effects model, and the results showed that the NRS2002 score could not be used as a predictor of risk factors for the development of refeeding syndromes in acutely ill patients [WMD = 0.68, 95% CI (−0.41, 1.77), *p* = 0.22].

**Supporting Information 10** Figure S10: Forest plot of SOFA score in relation to refeeding syndrome in acutely ill patients. Six studies [12, 13, 21–23, 25] reported the SOFA score, of which four [12, 13, 21, 23] had consistent data types for SOFA scores, and the meta‐analysis showed no heterogeneity (*I* ^2^ = 0%, *p* < 0.01), so the analysis was conducted using the fixed‐effects model, and the results showed that the difference was statistically significant [WMD = 1.87, 95% CI (1.50, 2.25), *p* < 0.01], suggesting that the SOFA score can be used as a risk factor for predicting the occurrence of refeeding syndrome in acutely ill patients.

**Supporting Information 11** Figure S11: Forest plot of the relationship between age and refeeding syndrome in acutely ill patients. Less heterogeneity between studies [12, 13, 15, 20, 24] (*I* ^2^ = 12%, *p* = 0.34), so the analysis was performed using a fixed‐effects model, and the results showed that the difference was statistically significant [WMD = 8.67, 95% CI (7.14, 10.19), *p* < 0.01], suggesting that age can be used as a predictor of risk for the development of refeeding syndromes in patients with acute and critical illnesses.

**Supporting Information 12** Figure S12: Forest plot of the relationship between feeding initiation within 48 h of ICU admission and refeeding syndrome in acutely ill patients. No heterogeneity between the studies [12, 13, 17] (*I* ^2^ = 0%, *p* = 0.79), so a fixed‐effects model was used in the analysis, and the results showed a statistically significant difference [OR = 1.98, 95% CI (1.56, 2.51), *p* < 0.01], suggesting that the initiation of feeding within 48h in the ICU can be used as a risk factor for predicting the occurrence of refeeding syndrome in acutely ill patients.

**Supporting Information 13** Figure S13: Forest plot of the relationship between history of diabetes and refeeding syndrome in acutely ill patients. Three studies [14, 16, 19] reported a history of diabetes, and the meta‐analysis showed heterogeneity among the studies (*I* ^2^ = 89%, *p* < 0.01). Therefore, the analysis was carried out using the random‐effects model, and the results showed that the difference was statistically significant [OR = 2.53, 95% CI (1.18, 5.44), *p* = 0.02], suggesting that a history of diabetes can be a risk factor for predicting the development of refeeding syndrome in acutely ill patients.

**Supporting Information 14** Table S1: Comparison table of risk factors in this study and NICE/ASPEN guidelines. This table is designed to compare the consistency between the RFS‐related risk factors identified in this study and those specified in two international authoritative guidelines. The table details the consistency level of each risk factor with the respective guidelines, supplemented by key notes clarifying the clinical significance of the factors and differences in guideline focus.

**Supporting Information 15** Table S2: Subgroup analysis. Subgroup analyses of the association between refeeding syndrome (RFS) development and serum phosphorus, albumin, prealbumin levels, and APACHE II scores in critically ill patients.

**Supporting Information 16** Table S3: Leave‐one‐out sensitivity analysis. This table presents the results of leave‐one‐out sensitivity analysis examining the robustness of our meta‐analysis findings. The analysis was performed by systematically excluding each included study one at a time to evaluate their individual influence on the pooled effect estimates.

## Data Availability

The data that support the findings of this study are available from the corresponding author upon reasonable request.
