# Supplementary material for: Meta‐Analysis of Refeeding Syndrome in Predicting the Risk of Occurrence in Critically Ill Patients
Source: J Nutr Metab. 2026 Feb 18;2026:6660254. doi: 10.1155/jnme/6660254 (PMC12917335; doi:10.1155/jnme/6660254)
Supplement: Supplementary file 1 — Supporting Information 1 Figure S1: Forest plot of baseline serum phosphate in relation to refeeding syndrome in acutely ill patients. Six studies [8–11, 18, 23] reported serum phosphorus levels (I 2 = 90%, p < 0.01), so the analysis was performed using a random‐effects model, and the results showed that the difference was statistically significant [WMD = −0.10, 95% CI (−0.19, −0.01), p = 0.03], suggesting that serum phosphorus levels can be used as a risk factor for predicting the occurrence of refeeding syndrome in acutely ill patients. [file JNME-2026-6660254-s015.pptx]

## Slide 1
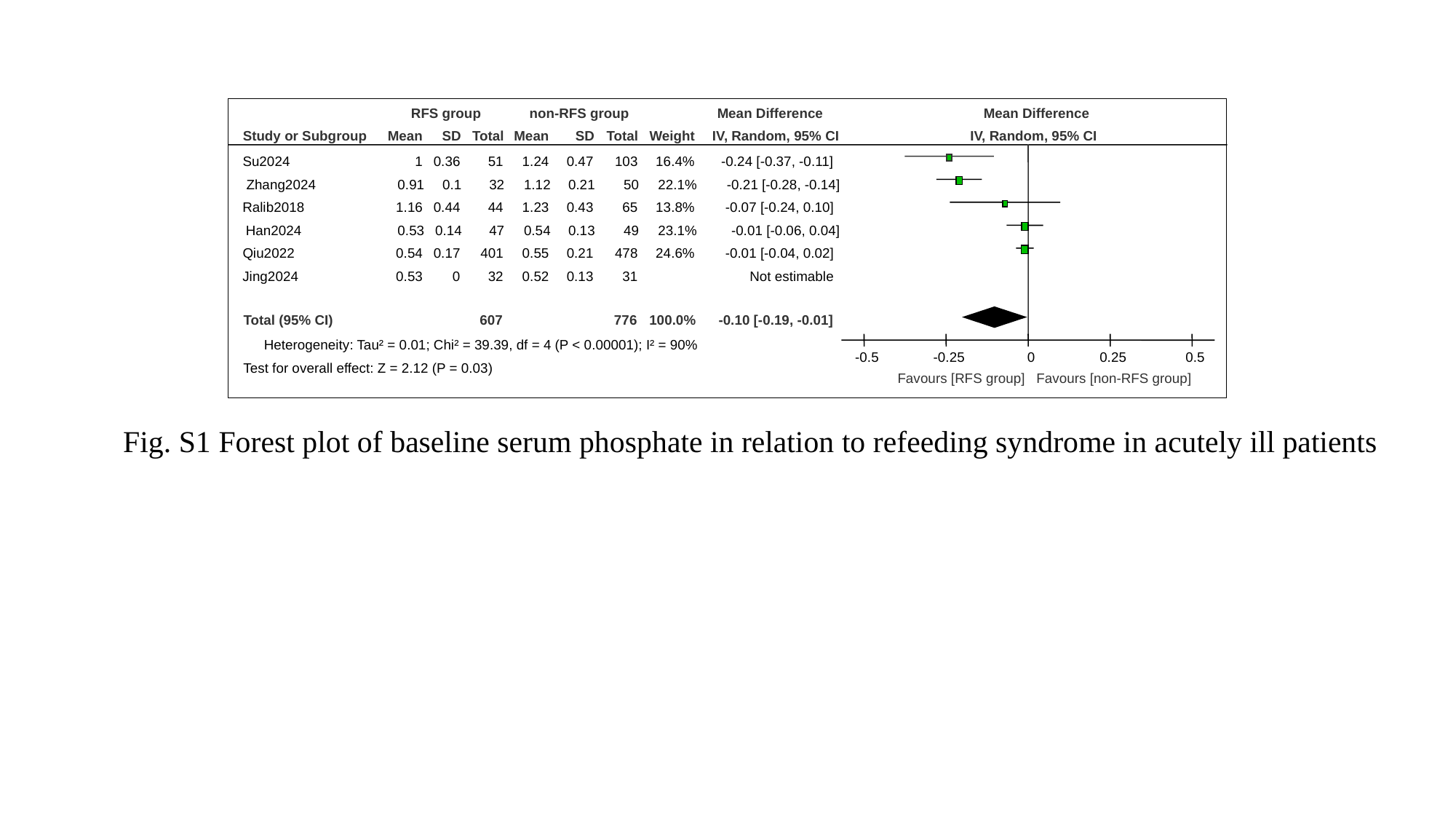

RFS group
non-RFS group
Mean Difference
Mean Difference
Study or Subgroup
Mean
SD
Total
Mean
SD
Total
Weight
IV, Random, 95% CI
IV, Random, 95% CI
Su2024
1
0.36
51
1.24
0.47
103
16.4%
-0.24 [-0.37, -0.11]
Zhang2024
0.91
0.1
32
1.12
0.21
50
22.1%
-0.21 [-0.28, -0.14]
Ralib2018
1.16
0.44
44
1.23
0.43
65
13.8%
-0.07 [-0.24, 0.10]
Han2024
0.53
0.14
47
0.54
0.13
49
23.1%
-0.01 [-0.06, 0.04]
Qiu2022
0.54
0.17
401
0.55
0.21
478
24.6%
-0.01 [-0.04, 0.02]
Jing2024
0.53
0
32
0.52
0.13
31
Not estimable
Total (95% CI)
607
776
100.0%
-0.10 [-0.19, -0.01]
Heterogeneity: Tau² = 0.01; Chi² = 39.39, df = 4 (P < 0.00001); I² = 90%
-0.5
-0.25
0
0.25
0.5
Test for overall effect: Z = 2.12 (P = 0.03)
Favours [RFS group]
Favours [non-RFS group]
Fig. S1 Forest plot of baseline serum phosphate in relation to refeeding syndrome in acutely ill patients
