# Supplementary material for: Meta‐Analysis of Refeeding Syndrome in Predicting the Risk of Occurrence in Critically Ill Patients
Source: J Nutr Metab. 2026 Feb 18;2026:6660254. doi: 10.1155/jnme/6660254 (PMC12917335; doi:10.1155/jnme/6660254)
Supplement: Supplementary file 2 — Supporting Information 2 Figure S2: Forest plot of baseline serum magnesium in relation to refeeding syndrome in acutely ill patients. Four studies [9, 11, 18, 23] reported serum magnesium levels (I 2 = 0%, p = 0.92), so the analysis was performed using a fixed‐effects model, and the results showed that serum magnesium level was not a predictor of risk factors for the development of refeeding syndromes in patients with acute and critical illnesses [WMD = −0.01, 95% CI (−0.04, 0.02), p = 0.54]. [file JNME-2026-6660254-s014.pptx]

## Slide 1
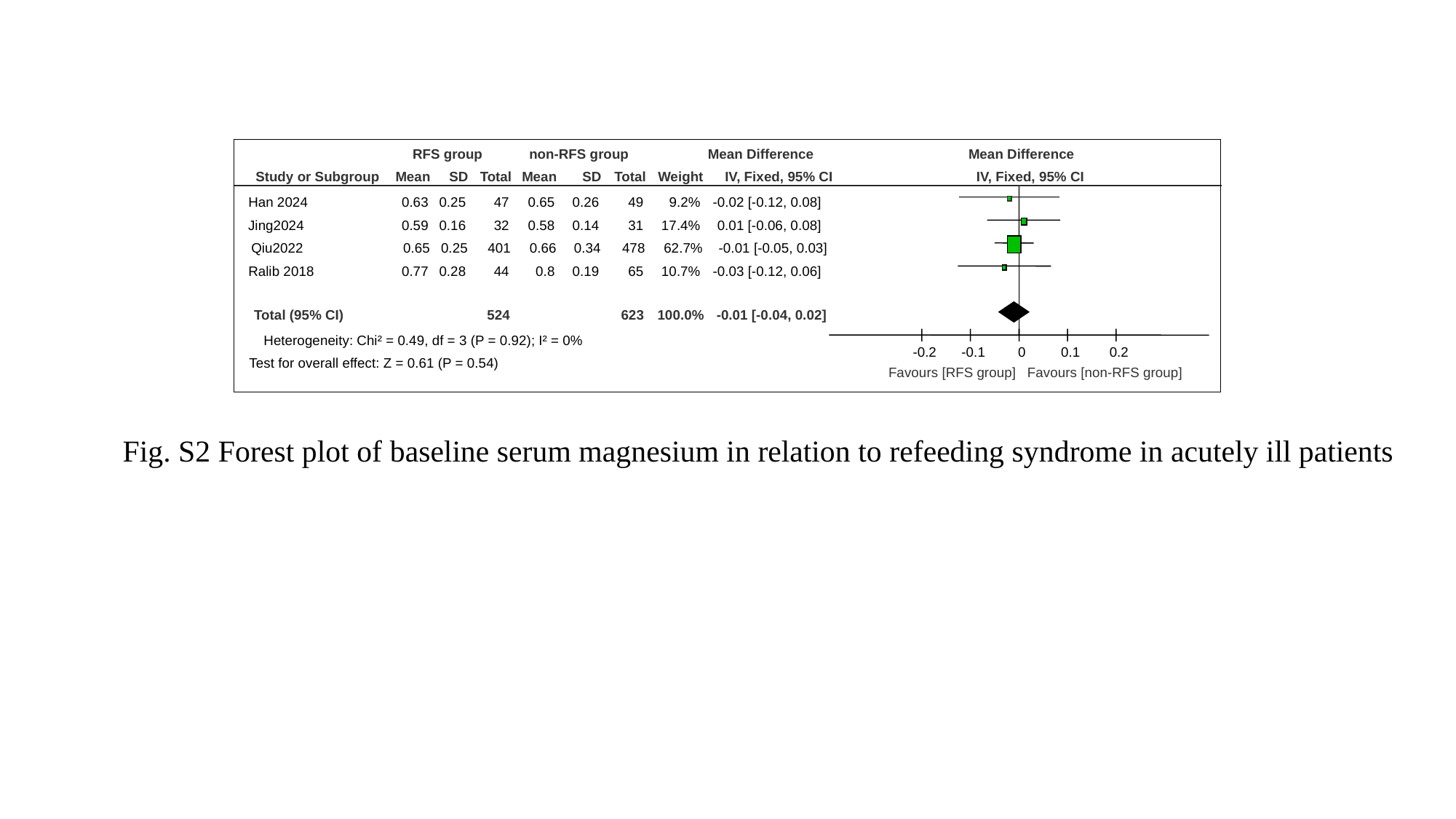

RFS group
non-RFS group
Mean Difference
Mean Difference
Study or Subgroup
Mean
SD
Total
Mean
SD
Total
Weight
IV, Fixed, 95% CI
IV, Fixed, 95% CI
Han 2024
0.63
0.25
47
0.65
0.26
49
9.2%
-0.02 [-0.12, 0.08]
Jing2024
0.59
0.16
32
0.58
0.14
31
17.4%
0.01 [-0.06, 0.08]
Qiu2022
0.65
0.25
401
0.66
0.34
478
62.7%
-0.01 [-0.05, 0.03]
Ralib 2018
0.77
0.28
44
0.8
0.19
65
10.7%
-0.03 [-0.12, 0.06]
Total (95% CI)
524
623
100.0%
-0.01 [-0.04, 0.02]
Heterogeneity: Chi² = 0.49, df = 3 (P = 0.92); I² = 0%
-0.2
-0.1
0
0.1
0.2
Test for overall effect: Z = 0.61 (P = 0.54)
Favours [RFS group]
Favours [non-RFS group]
Fig. S2 Forest plot of baseline serum magnesium in relation to refeeding syndrome in acutely ill patients
