# Supplementary material for: Meta‐Analysis of Refeeding Syndrome in Predicting the Risk of Occurrence in Critically Ill Patients
Source: J Nutr Metab. 2026 Feb 18;2026:6660254. doi: 10.1155/jnme/6660254 (PMC12917335; doi:10.1155/jnme/6660254)
Supplement: Supplementary file 3 — Supporting Information 3 Figure S3: Forest plot of baseline serum potassium in relation to refeeding syndrome in acutely ill patients. Six studies [9–11, 18, 21, 23] reported serum potassium levels (I 2 = 11%, p = 0.34), so the analysis was carried out by using the fixed‐effects model, and the results showed that serum potassium level was not a predictor of risk factors for the development of refeeding syndromes in patients with acute and critical illnesses [WMD = −0.02, 95% CI (−0.06, 0.02), p = 0.28]. [file JNME-2026-6660254-s013.pptx]

## Slide 1
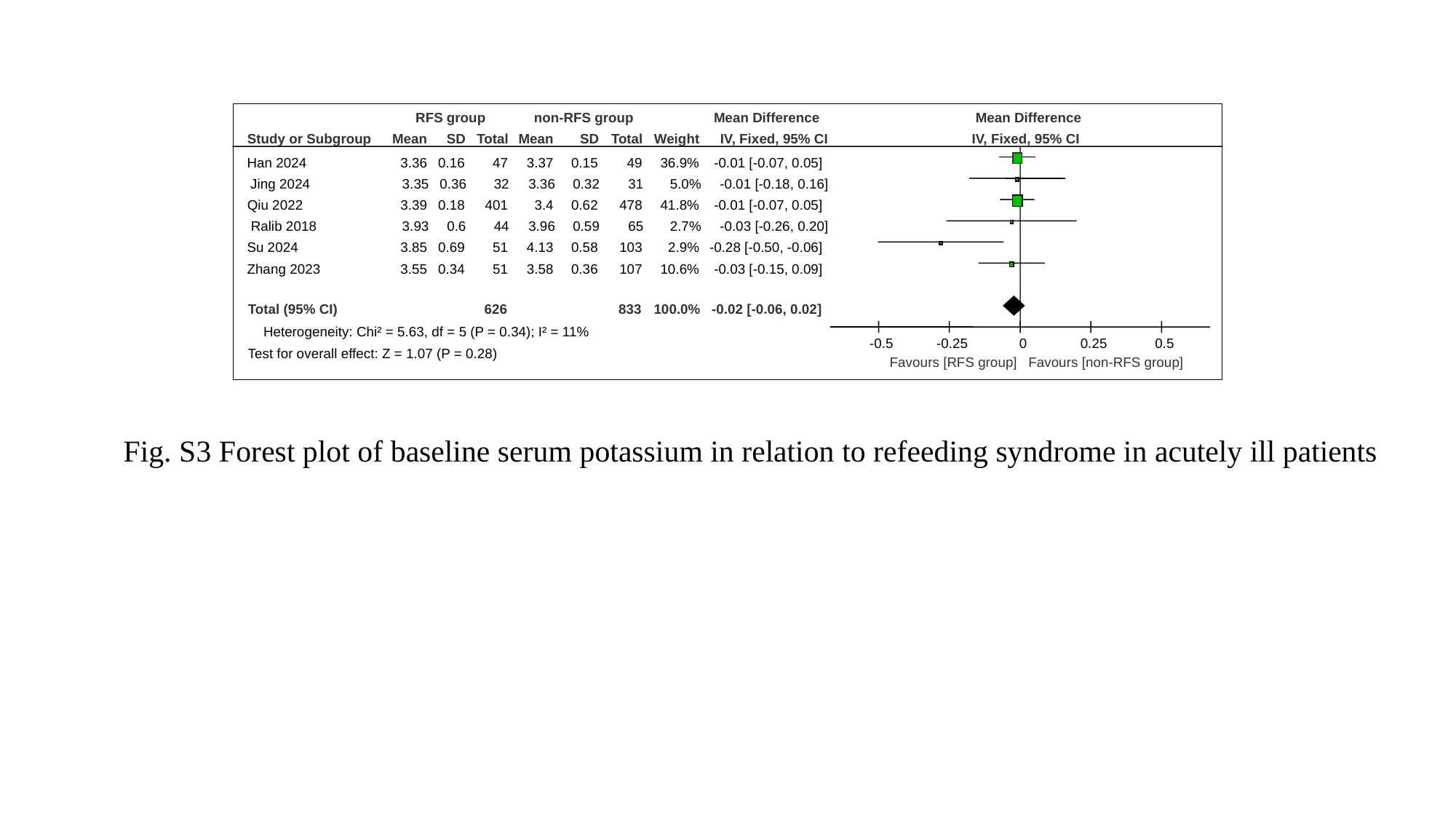

RFS group
non-RFS group
Mean Difference
Mean Difference
Study or Subgroup
Mean
SD
Total
Mean
SD
Total
Weight
IV, Fixed, 95% CI
IV, Fixed, 95% CI
Han 2024
3.36
0.16
47
3.37
0.15
49
36.9%
-0.01 [-0.07, 0.05]
Jing 2024
3.35
0.36
32
3.36
0.32
31
5.0%
-0.01 [-0.18, 0.16]
Qiu 2022
3.39
0.18
401
3.4
0.62
478
41.8%
-0.01 [-0.07, 0.05]
Ralib 2018
3.93
0.6
44
3.96
0.59
65
2.7%
-0.03 [-0.26, 0.20]
Su 2024
3.85
0.69
51
4.13
0.58
103
2.9%
-0.28 [-0.50, -0.06]
Zhang 2023
3.55
0.34
51
3.58
0.36
107
10.6%
-0.03 [-0.15, 0.09]
Total (95% CI)
626
833
100.0%
-0.02 [-0.06, 0.02]
Heterogeneity: Chi² = 5.63, df = 5 (P = 0.34); I² = 11%
-0.5
-0.25
0
0.25
0.5
Test for overall effect: Z = 1.07 (P = 0.28)
Favours [RFS group]
Favours [non-RFS group]
Fig. S3 Forest plot of baseline serum potassium in relation to refeeding syndrome in acutely ill patients
