# Supplementary material for: Meta‐Analysis of Refeeding Syndrome in Predicting the Risk of Occurrence in Critically Ill Patients
Source: J Nutr Metab. 2026 Feb 18;2026:6660254. doi: 10.1155/jnme/6660254 (PMC12917335; doi:10.1155/jnme/6660254)
Supplement: Supplementary file 4 — Supporting Information 4 Figure S4: Forest plot of baseline serum albumin in relation to refeeding syndrome in acutely ill patients. Ten studies [9, 11, 13–18, 21, 23] reported serum albumin levels, of which seven [9, 11, 13, 15, 18, 21, 23] had consistent data types (I 2 = 93%, p < 0.01), so the analysis was performed using a random‐effects model, and the results showed that the difference was statistically significant [WMD = −2.08, 95% CI (−3.81, −0.36), p = 0.02], suggesting that serum albumin level can be used as a risk factor for predicting the occurrence of refeeding syndrome in acutely ill patients. [file JNME-2026-6660254-s012.pptx]

## Slide 1
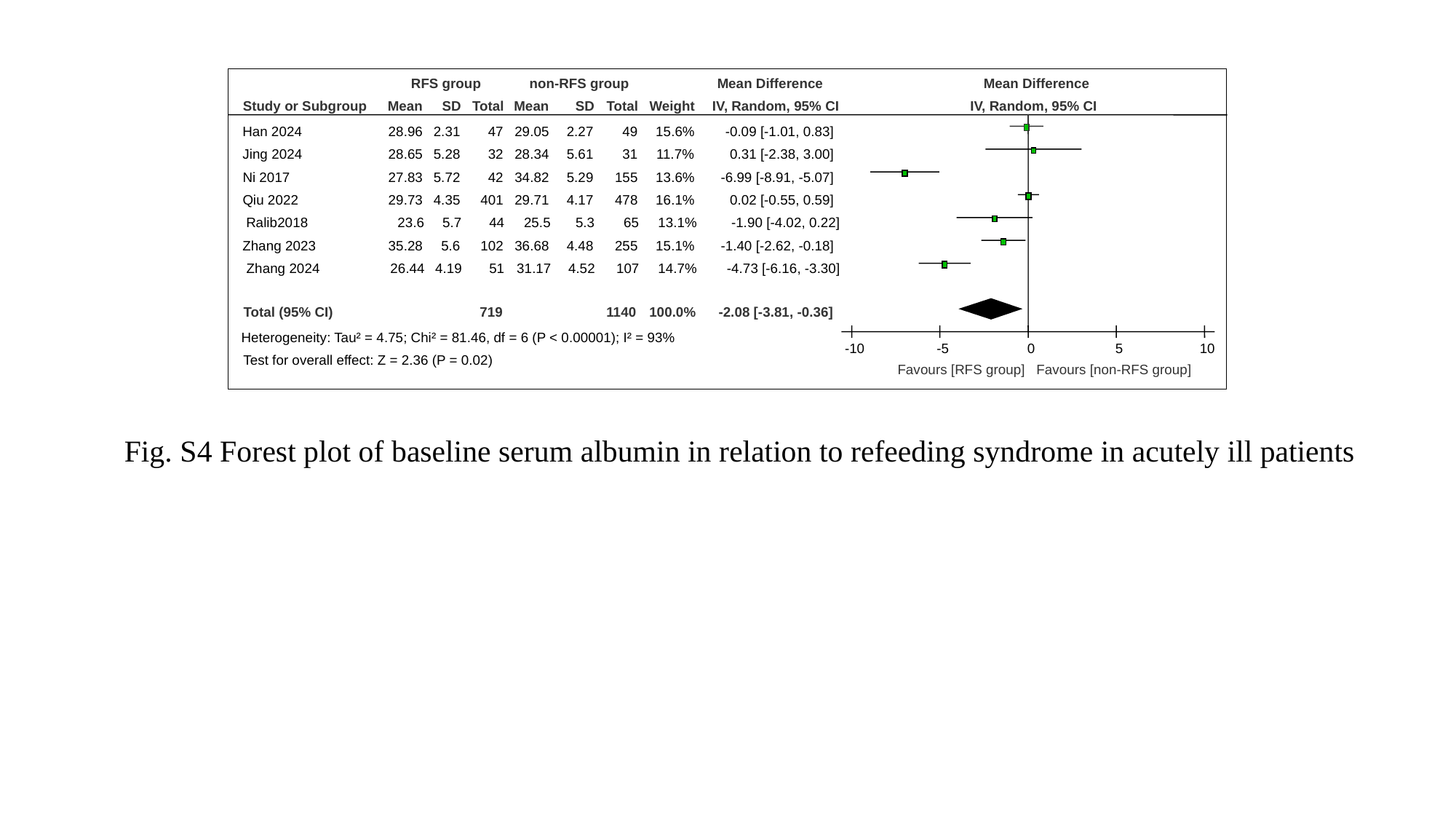

RFS group
non-RFS group
Mean Difference
Mean Difference
Study or Subgroup
Mean
SD
Total
Mean
SD
Total
Weight
IV, Random, 95% CI
IV, Random, 95% CI
Han 2024
28.96
2.31
47
29.05
2.27
49
15.6%
-0.09 [-1.01, 0.83]
Jing 2024
28.65
5.28
32
28.34
5.61
31
11.7%
0.31 [-2.38, 3.00]
Ni 2017
27.83
5.72
42
34.82
5.29
155
13.6%
-6.99 [-8.91, -5.07]
Qiu 2022
29.73
4.35
401
29.71
4.17
478
16.1%
0.02 [-0.55, 0.59]
Ralib2018
23.6
5.7
44
25.5
5.3
65
13.1%
-1.90 [-4.02, 0.22]
Zhang 2023
35.28
5.6
102
36.68
4.48
255
15.1%
-1.40 [-2.62, -0.18]
Zhang 2024
26.44
4.19
51
31.17
4.52
107
14.7%
-4.73 [-6.16, -3.30]
Total (95% CI)
719
1140
100.0%
-2.08 [-3.81, -0.36]
Heterogeneity: Tau² = 4.75; Chi² = 81.46, df = 6 (P < 0.00001); I² = 93%
-10
-5
0
5
10
Test for overall effect: Z = 2.36 (P = 0.02)
Favours [RFS group]
Favours [non-RFS group]
Fig. S4 Forest plot of baseline serum albumin in relation to refeeding syndrome in acutely ill patients
