# Supplementary material for: Meta‐Analysis of Refeeding Syndrome in Predicting the Risk of Occurrence in Critically Ill Patients
Source: J Nutr Metab. 2026 Feb 18;2026:6660254. doi: 10.1155/jnme/6660254 (PMC12917335; doi:10.1155/jnme/6660254)
Supplement: Supplementary file 5 — Supporting Information 5 Figure S5: Forest plot of baseline serum prealbumin in relation to refeeding syndrome in acutely ill patients. Nine studies [9, 11, 13–19] reported serum prealbumin levels, of which five [9, 11, 13, 15, 18] had consistent data types (I 2 = 96%, p < 0.01), so the analysis was performed using a random‐effects model, and the results showed that serum prealbumin level was not a predictor of risk factors for the development of refeeding syndromes in patients with acute and critical illnesses [WMD = −15.37, 95% CI (−33.00, 2.27), p = 0.09]. [file JNME-2026-6660254-s016.pptx]

## Slide 1
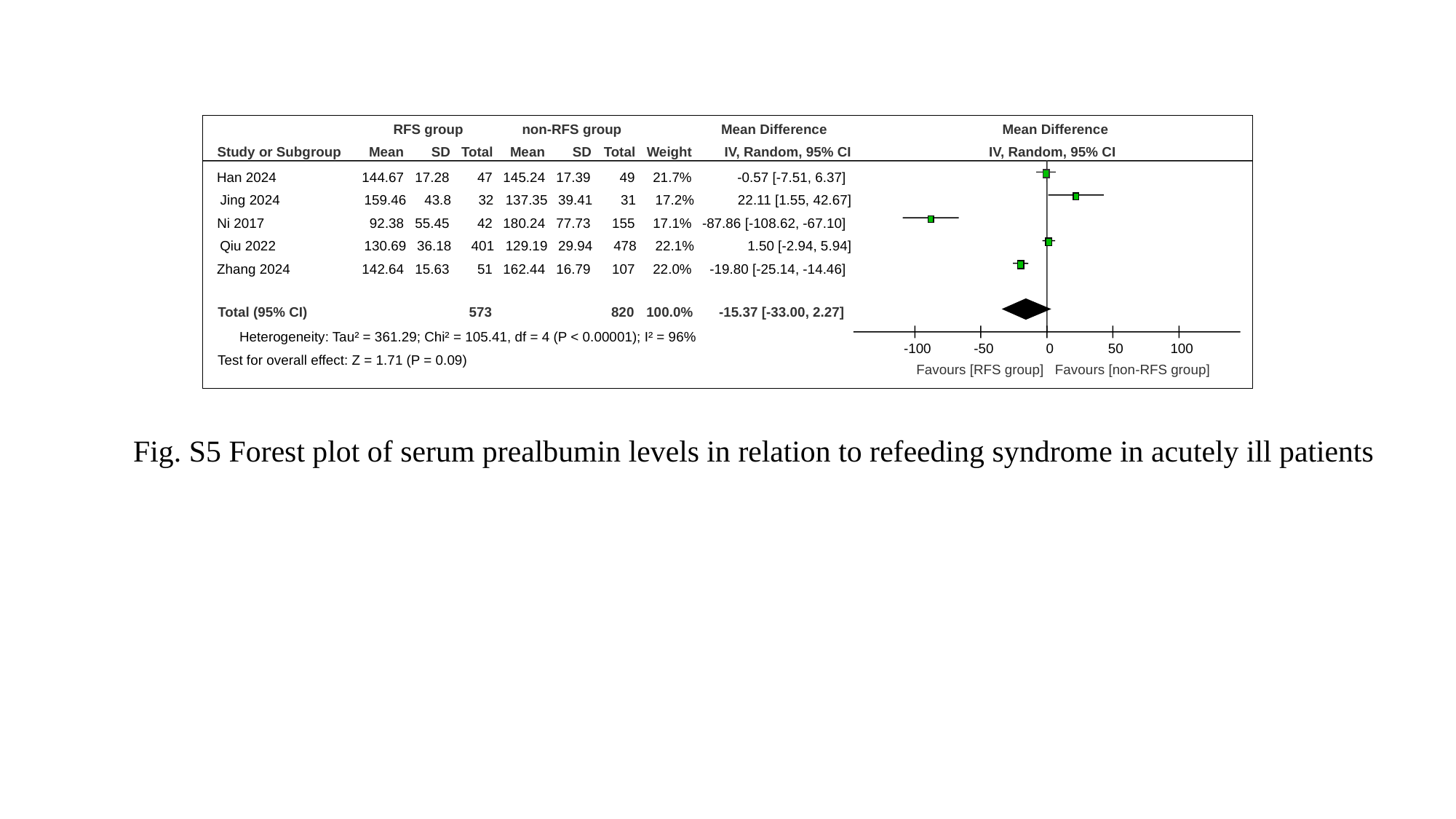

RFS group
non-RFS group
Mean Difference
Mean Difference
Study or Subgroup
Mean
SD
Total
Mean
SD
Total
Weight
IV, Random, 95% CI
IV, Random, 95% CI
Han 2024
144.67
17.28
47
145.24
17.39
49
21.7%
-0.57 [-7.51, 6.37]
Jing 2024
159.46
43.8
32
137.35
39.41
31
17.2%
22.11 [1.55, 42.67]
Ni 2017
92.38
55.45
42
180.24
77.73
155
17.1%
-87.86 [-108.62, -67.10]
Qiu 2022
130.69
36.18
401
129.19
29.94
478
22.1%
1.50 [-2.94, 5.94]
Zhang 2024
142.64
15.63
51
162.44
16.79
107
22.0%
-19.80 [-25.14, -14.46]
Total (95% CI)
573
820
100.0%
-15.37 [-33.00, 2.27]
Heterogeneity: Tau² = 361.29; Chi² = 105.41, df = 4 (P < 0.00001); I² = 96%
-100
-50
0
50
100
Test for overall effect: Z = 1.71 (P = 0.09)
Favours [RFS group]
Favours [non-RFS group]
Fig. S5 Forest plot of serum prealbumin levels in relation to refeeding syndrome in acutely ill patients
