# Supplementary material for: Meta‐Analysis of Refeeding Syndrome in Predicting the Risk of Occurrence in Critically Ill Patients
Source: J Nutr Metab. 2026 Feb 18;2026:6660254. doi: 10.1155/jnme/6660254 (PMC12917335; doi:10.1155/jnme/6660254)
Supplement: Supplementary file 6 — Supporting Information 6 Figure S6: Forest plot of daily protein intake in relation to refeeding syndrome in acutely ill patients. Six studies [8, 12–14, 16, 24] reported daily protein intake, of which two [12, 13] had consistent data types (I 2 = 0%, p = 0.87), so the analysis was carried out using a fixed‐effects model, and the results showed that the difference was statistically significant [WMD = 0.23, 95% CI (0.17, 0.28), p < 0.01], suggesting that daily protein intake can be used as a risk factor for predicting the development of refeeding syndrome in acutely ill patients. [file JNME-2026-6660254-s010.pptx]

## Slide 1
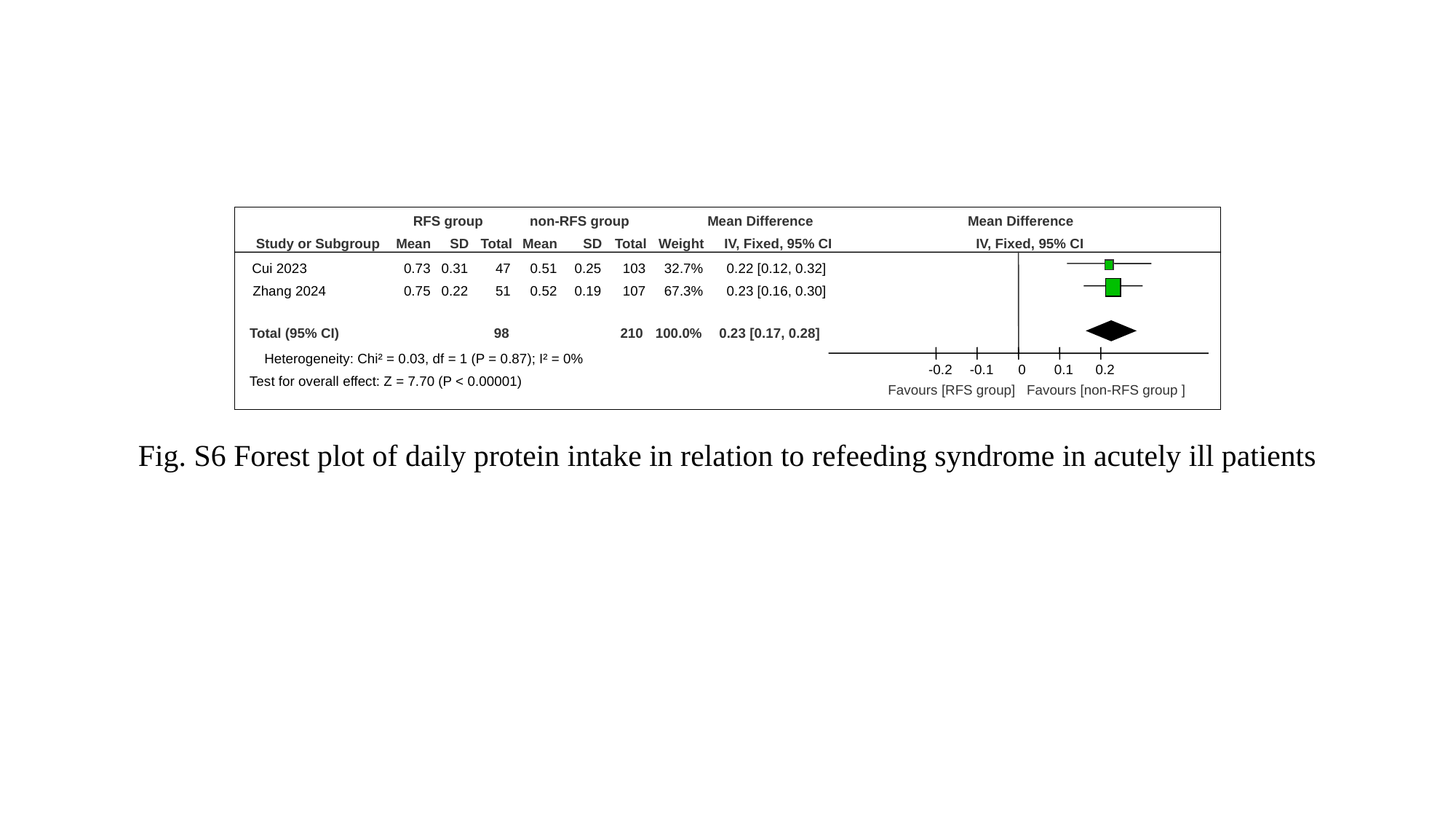

RFS group
non-RFS group
Mean Difference
Mean Difference
Study or Subgroup
Mean
SD
Total
Mean
SD
Total
Weight
IV, Fixed, 95% CI
IV, Fixed, 95% CI
Cui 2023
0.73
0.31
47
0.51
0.25
103
32.7%
0.22 [0.12, 0.32]
Zhang 2024
0.75
0.22
51
0.52
0.19
107
67.3%
0.23 [0.16, 0.30]
Total (95% CI)
98
210
100.0%
0.23 [0.17, 0.28]
Heterogeneity: Chi² = 0.03, df = 1 (P = 0.87); I² = 0%
-0.2
-0.1
0
0.1
0.2
Test for overall effect: Z = 7.70 (P < 0.00001)
Favours [RFS group]
Favours [non-RFS group ]
Fig. S6 Forest plot of daily protein intake in relation to refeeding syndrome in acutely ill patients
