# Supplementary material for: Meta‐Analysis of Refeeding Syndrome in Predicting the Risk of Occurrence in Critically Ill Patients
Source: J Nutr Metab. 2026 Feb 18;2026:6660254. doi: 10.1155/jnme/6660254 (PMC12917335; doi:10.1155/jnme/6660254)
Supplement: Supplementary file 7 — Supporting Information 7 Figure S7: Forest plot of daily calorie intake in relation to refeeding syndrome in acutely ill patients. No heterogeneity between the studies [14, 16] (I 2 = 0%, p = 0.61), so a fixed‐effects model was used in the analysis, and the results showed a statistically significant difference [OR = 0.35, 95% CI (0.25, 0.49), p < 0.01], suggesting that daily calorie intake can be used as a risk factor for predicting the occurrence of refeeding syndrome in acutely ill patients. [file JNME-2026-6660254-s009.pptx]

## Slide 1
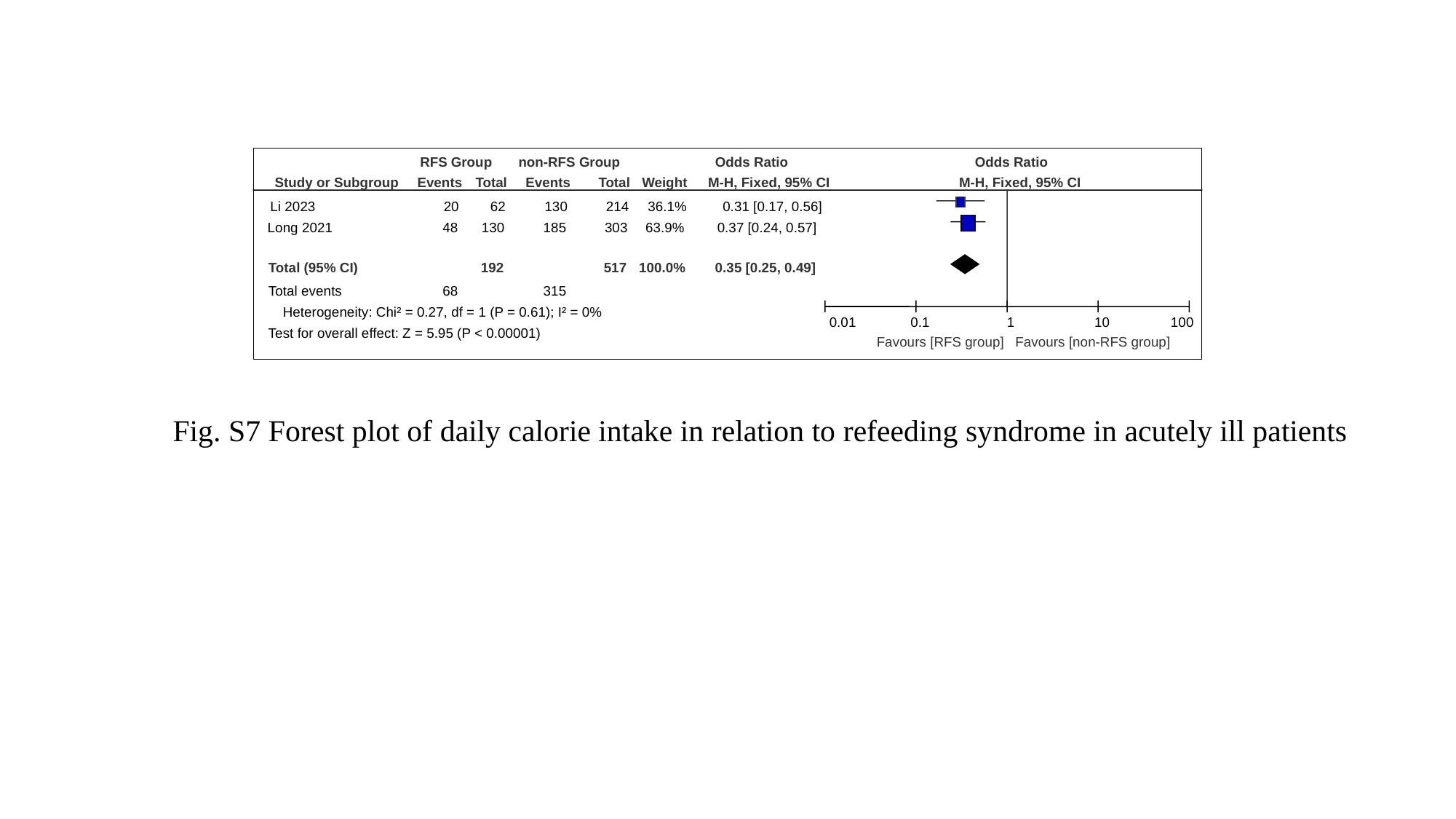

RFS Group
non-RFS Group
Odds Ratio
Odds Ratio
Study or Subgroup
Events
Total
Events
Total
Weight
M-H, Fixed, 95% CI
M-H, Fixed, 95% CI
Li 2023
20
62
130
214
36.1%
0.31 [0.17, 0.56]
Long 2021
48
130
185
303
63.9%
0.37 [0.24, 0.57]
Total (95% CI)
192
517
100.0%
0.35 [0.25, 0.49]
Total events
68
315
Heterogeneity: Chi² = 0.27, df = 1 (P = 0.61); I² = 0%
0.01
0.1
1
10
100
Test for overall effect: Z = 5.95 (P < 0.00001)
Favours [RFS group]
Favours [non-RFS group]
Fig. S7 Forest plot of daily calorie intake in relation to refeeding syndrome in acutely ill patients
