# Supplementary material for: Meta‐Analysis of Refeeding Syndrome in Predicting the Risk of Occurrence in Critically Ill Patients
Source: J Nutr Metab. 2026 Feb 18;2026:6660254. doi: 10.1155/jnme/6660254 (PMC12917335; doi:10.1155/jnme/6660254)
Supplement: Supplementary file 8 — Supporting Information 8 Figure S8: Forest plot of APACHEII score in relation to refeeding syndrome in acutely ill patients. Six studies [10, 13, 17–19, 21, 25] reported the APACHE II score, and the meta‐analysis results showed heterogeneity between studies (I 2 = 89%, p < 0.01), so the analysis was carried out using the random‐effects model, and the results showed that the difference was statistically significant [WMD = 2.65, 95% CI (1.22, 4.08), p < 0.01], suggesting that the APACHE II score can be used as a risk factor for predicting the occurrence of refeeding syndrome in acutely ill patients. [file JNME-2026-6660254-s001.pptx]

## Slide 1
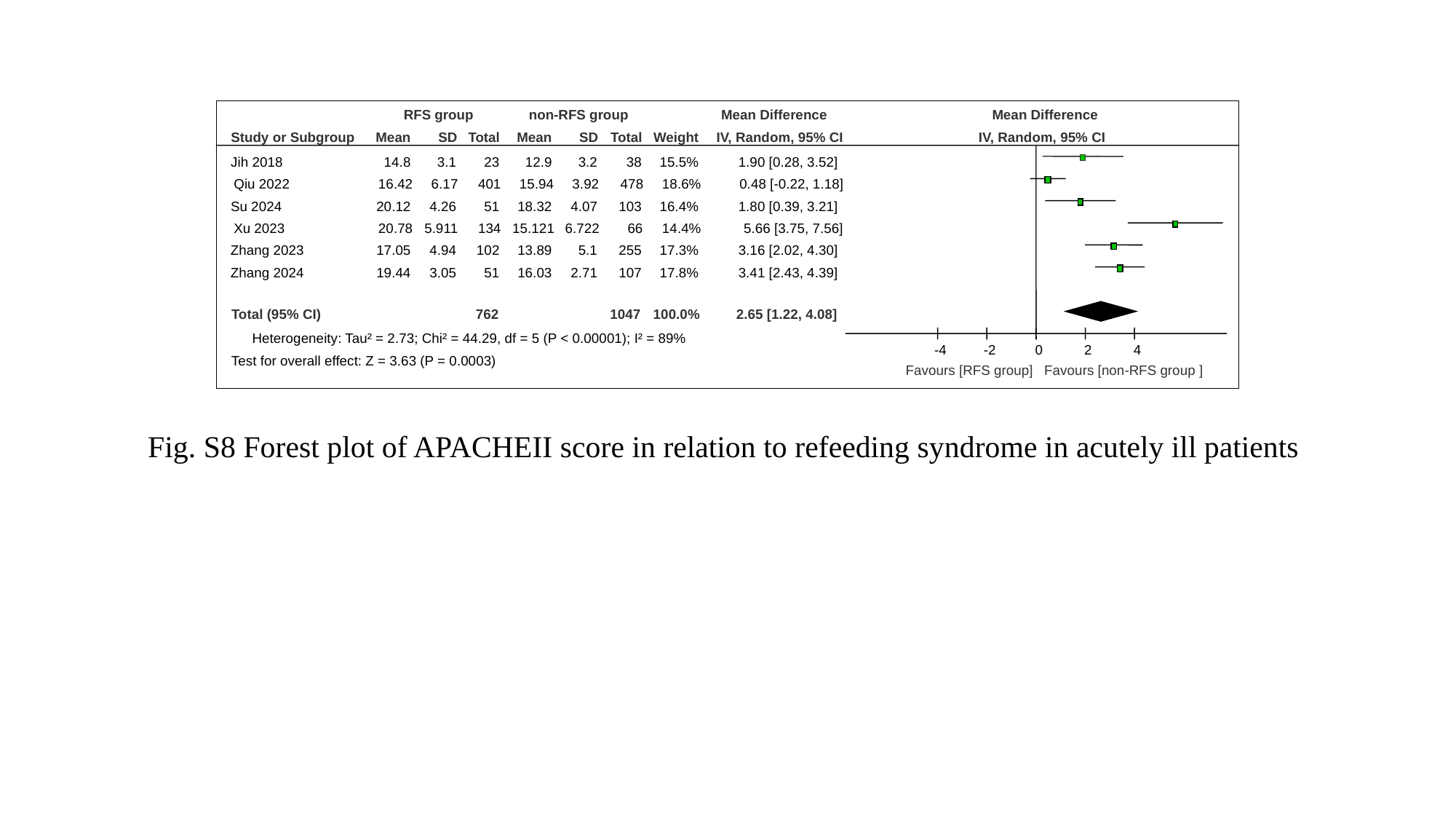

RFS group
non-RFS group
Mean Difference
Mean Difference
Study or Subgroup
Mean
SD
Total
Mean
SD
Total
Weight
IV, Random, 95% CI
IV, Random, 95% CI
Jih 2018
14.8
3.1
23
12.9
3.2
38
15.5%
1.90 [0.28, 3.52]
Qiu 2022
16.42
6.17
401
15.94
3.92
478
18.6%
0.48 [-0.22, 1.18]
Su 2024
20.12
4.26
51
18.32
4.07
103
16.4%
1.80 [0.39, 3.21]
Xu 2023
20.78
5.911
134
15.121
6.722
66
14.4%
5.66 [3.75, 7.56]
Zhang 2023
17.05
4.94
102
13.89
5.1
255
17.3%
3.16 [2.02, 4.30]
Zhang 2024
19.44
3.05
51
16.03
2.71
107
17.8%
3.41 [2.43, 4.39]
Total (95% CI)
762
1047
100.0%
2.65 [1.22, 4.08]
Heterogeneity: Tau² = 2.73; Chi² = 44.29, df = 5 (P < 0.00001); I² = 89%
-4
-2
0
2
4
Test for overall effect: Z = 3.63 (P = 0.0003)
Favours [RFS group]
Favours [non-RFS group ]
Fig. S8 Forest plot of APACHEII score in relation to refeeding syndrome in acutely ill patients
