# Supplementary material for: Meta‐Analysis of Refeeding Syndrome in Predicting the Risk of Occurrence in Critically Ill Patients
Source: J Nutr Metab. 2026 Feb 18;2026:6660254. doi: 10.1155/jnme/6660254 (PMC12917335; doi:10.1155/jnme/6660254)
Supplement: Supplementary file 9 — Supporting Information 9 Figure S9: Forest plot of NRS2002 score in relation to refeeding syndrome in acutely ill patients. Five studies [10, 13, 14, 16, 19] reported the NRS2002 score, of which three [10, 13, 19] had consistent data types, and the meta‐analysis showed heterogeneity between studies (I 2 = 97%, p < 0.01), so the analysis was carried out using the random‐effects model, and the results showed that the NRS2002 score could not be used as a predictor of risk factors for the development of refeeding syndromes in acutely ill patients [WMD = 0.68, 95% CI (−0.41, 1.77), p = 0.22]. [file JNME-2026-6660254-s002.pptx]

## Slide 1
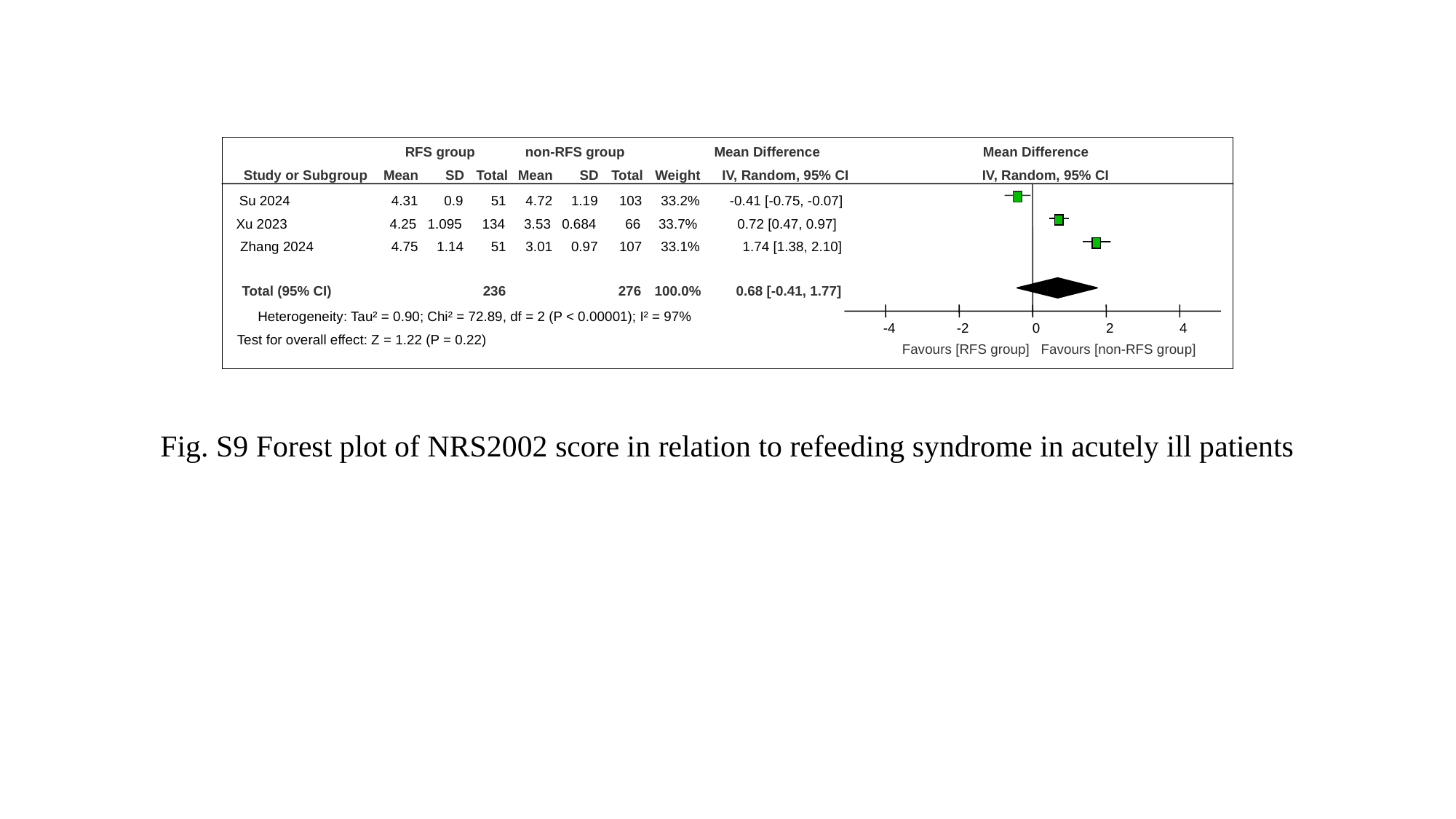

RFS group
non-RFS group
Mean Difference
Mean Difference
Study or Subgroup
Mean
SD
Total
Mean
SD
Total
Weight
IV, Random, 95% CI
IV, Random, 95% CI
Su 2024
4.31
0.9
51
4.72
1.19
103
33.2%
-0.41 [-0.75, -0.07]
Xu 2023
4.25
1.095
134
3.53
0.684
66
33.7%
0.72 [0.47, 0.97]
Zhang 2024
4.75
1.14
51
3.01
0.97
107
33.1%
1.74 [1.38, 2.10]
Total (95% CI)
236
276
100.0%
0.68 [-0.41, 1.77]
Heterogeneity: Tau² = 0.90; Chi² = 72.89, df = 2 (P < 0.00001); I² = 97%
-4
-2
0
2
4
Test for overall effect: Z = 1.22 (P = 0.22)
Favours [RFS group]
Favours [non-RFS group]
Fig. S9 Forest plot of NRS2002 score in relation to refeeding syndrome in acutely ill patients
