# Supplementary material for: Meta‐Analysis of Refeeding Syndrome in Predicting the Risk of Occurrence in Critically Ill Patients
Source: J Nutr Metab. 2026 Feb 18;2026:6660254. doi: 10.1155/jnme/6660254 (PMC12917335; doi:10.1155/jnme/6660254)
Supplement: Supplementary file 10 — Supporting Information 10 Figure S10: Forest plot of SOFA score in relation to refeeding syndrome in acutely ill patients. Six studies [12, 13, 21–23, 25] reported the SOFA score, of which four [12, 13, 21, 23] had consistent data types for SOFA scores, and the meta‐analysis showed no heterogeneity (I 2 = 0%, p < 0.01), so the analysis was conducted using the fixed‐effects model, and the results showed that the difference was statistically significant [WMD = 1.87, 95% CI (1.50, 2.25), p < 0.01], suggesting that the SOFA score can be used as a risk factor for predicting the occurrence of refeeding syndrome in acutely ill patients. [file JNME-2026-6660254-s003.pptx]

## Slide 1
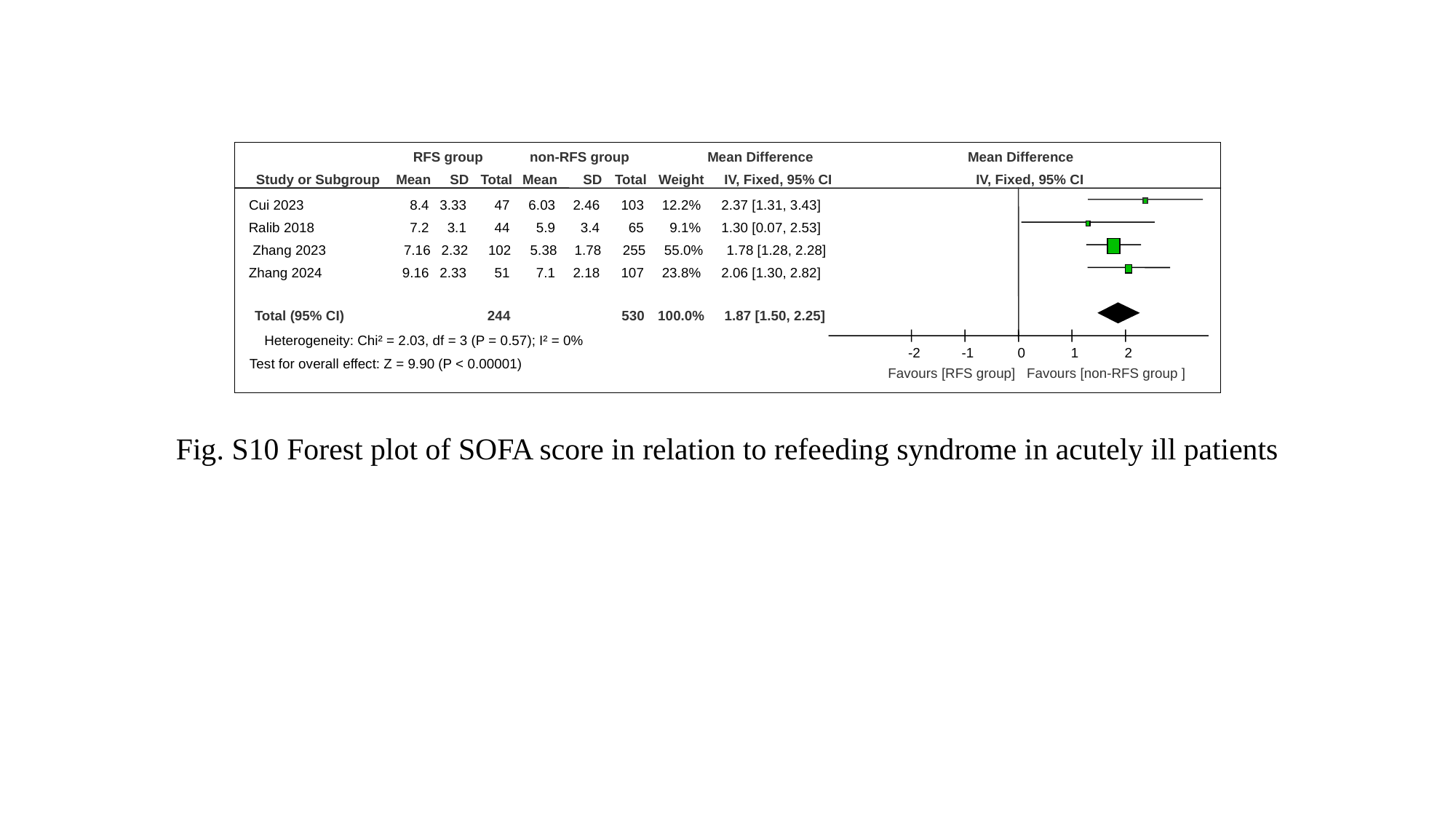

RFS group
non-RFS group
Mean Difference
Mean Difference
Study or Subgroup
Mean
SD
Total
Mean
SD
Total
Weight
IV, Fixed, 95% CI
IV, Fixed, 95% CI
Cui 2023
8.4
3.33
47
6.03
2.46
103
12.2%
2.37 [1.31, 3.43]
Ralib 2018
7.2
3.1
44
5.9
3.4
65
9.1%
1.30 [0.07, 2.53]
Zhang 2023
7.16
2.32
102
5.38
1.78
255
55.0%
1.78 [1.28, 2.28]
Zhang 2024
9.16
2.33
51
7.1
2.18
107
23.8%
2.06 [1.30, 2.82]
Total (95% CI)
244
530
100.0%
1.87 [1.50, 2.25]
Heterogeneity: Chi² = 2.03, df = 3 (P = 0.57); I² = 0%
-2
-1
0
1
2
Test for overall effect: Z = 9.90 (P < 0.00001)
Favours [RFS group]
Favours [non-RFS group ]
Fig. S10 Forest plot of SOFA score in relation to refeeding syndrome in acutely ill patients
