# Supplementary material for: Meta‐Analysis of Refeeding Syndrome in Predicting the Risk of Occurrence in Critically Ill Patients
Source: J Nutr Metab. 2026 Feb 18;2026:6660254. doi: 10.1155/jnme/6660254 (PMC12917335; doi:10.1155/jnme/6660254)
Supplement: Supplementary file 11 — Supporting Information 11 Figure S11: Forest plot of the relationship between age and refeeding syndrome in acutely ill patients. Less heterogeneity between studies [12, 13, 15, 20, 24] (I 2 = 12%, p = 0.34), so the analysis was performed using a fixed‐effects model, and the results showed that the difference was statistically significant [WMD = 8.67, 95% CI (7.14, 10.19), p < 0.01], suggesting that age can be used as a predictor of risk for the development of refeeding syndromes in patients with acute and critical illnesses. [file JNME-2026-6660254-s004.pptx]

## Slide 1
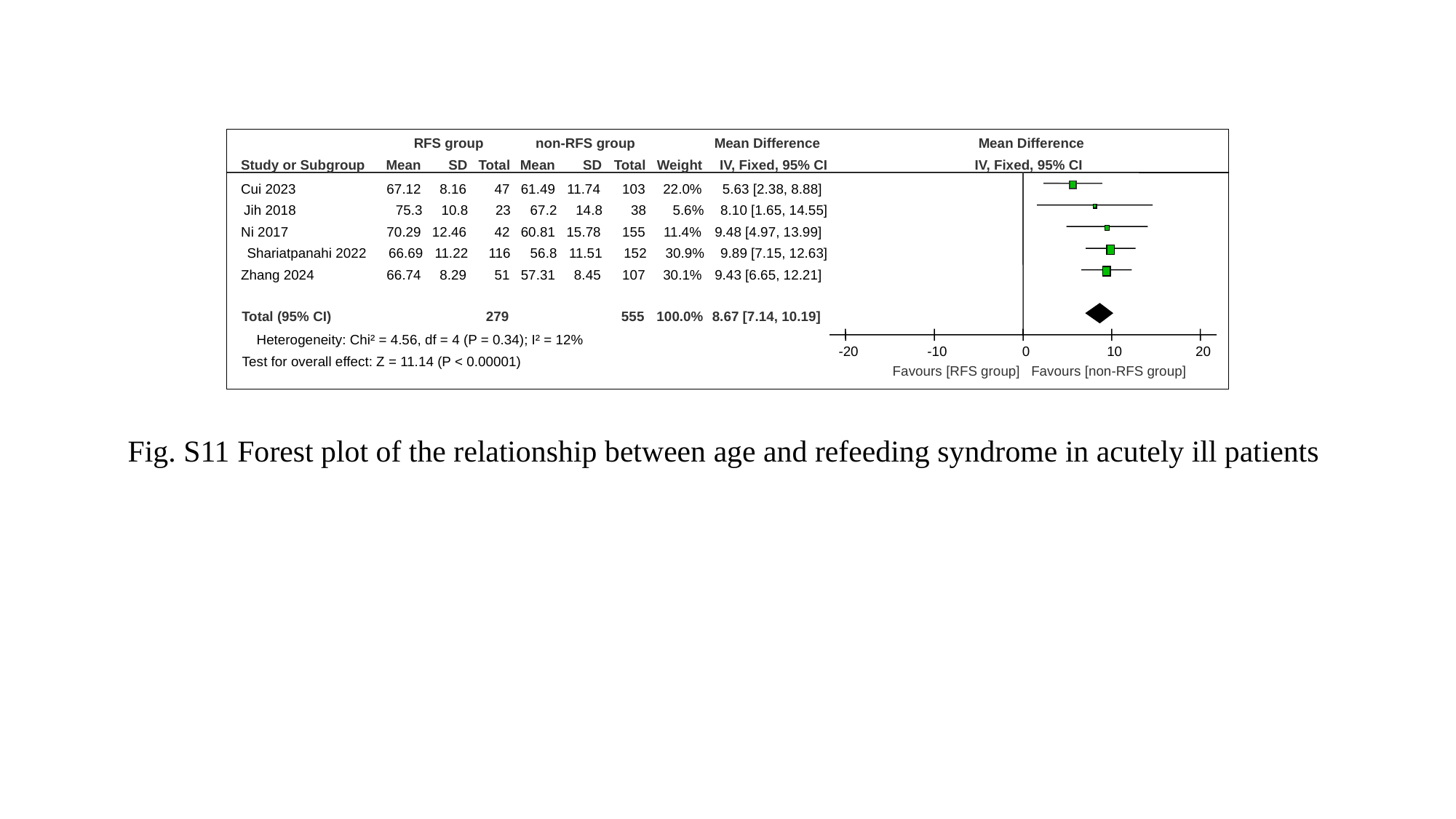

RFS group
non-RFS group
Mean Difference
Mean Difference
Study or Subgroup
Mean
SD
Total
Mean
SD
Total
Weight
IV, Fixed, 95% CI
IV, Fixed, 95% CI
Cui 2023
67.12
8.16
47
61.49
11.74
103
22.0%
5.63 [2.38, 8.88]
Jih 2018
75.3
10.8
23
67.2
14.8
38
5.6%
8.10 [1.65, 14.55]
Ni 2017
70.29
12.46
42
60.81
15.78
155
11.4%
9.48 [4.97, 13.99]
Shariatpanahi 2022
66.69
11.22
116
56.8
11.51
152
30.9%
9.89 [7.15, 12.63]
Zhang 2024
66.74
8.29
51
57.31
8.45
107
30.1%
9.43 [6.65, 12.21]
Total (95% CI)
279
555
100.0%
8.67 [7.14, 10.19]
Heterogeneity: Chi² = 4.56, df = 4 (P = 0.34); I² = 12%
-20
-10
0
10
20
Test for overall effect: Z = 11.14 (P < 0.00001)
Favours [RFS group]
Favours [non-RFS group]
Fig. S11 Forest plot of the relationship between age and refeeding syndrome in acutely ill patients
