# Supplementary material for: Meta‐Analysis of Refeeding Syndrome in Predicting the Risk of Occurrence in Critically Ill Patients
Source: J Nutr Metab. 2026 Feb 18;2026:6660254. doi: 10.1155/jnme/6660254 (PMC12917335; doi:10.1155/jnme/6660254)
Supplement: Supplementary file 12 — Supporting Information 12 Figure S12: Forest plot of the relationship between feeding initiation within 48 h of ICU admission and refeeding syndrome in acutely ill patients. No heterogeneity between the studies [12, 13, 17] (I 2 = 0%, p = 0.79), so a fixed‐effects model was used in the analysis, and the results showed a statistically significant difference [OR = 1.98, 95% CI (1.56, 2.51), p < 0.01], suggesting that the initiation of feeding within 48h in the ICU can be used as a risk factor for predicting the occurrence of refeeding syndrome in acutely ill patients. [file JNME-2026-6660254-s005.pptx]

## Slide 1
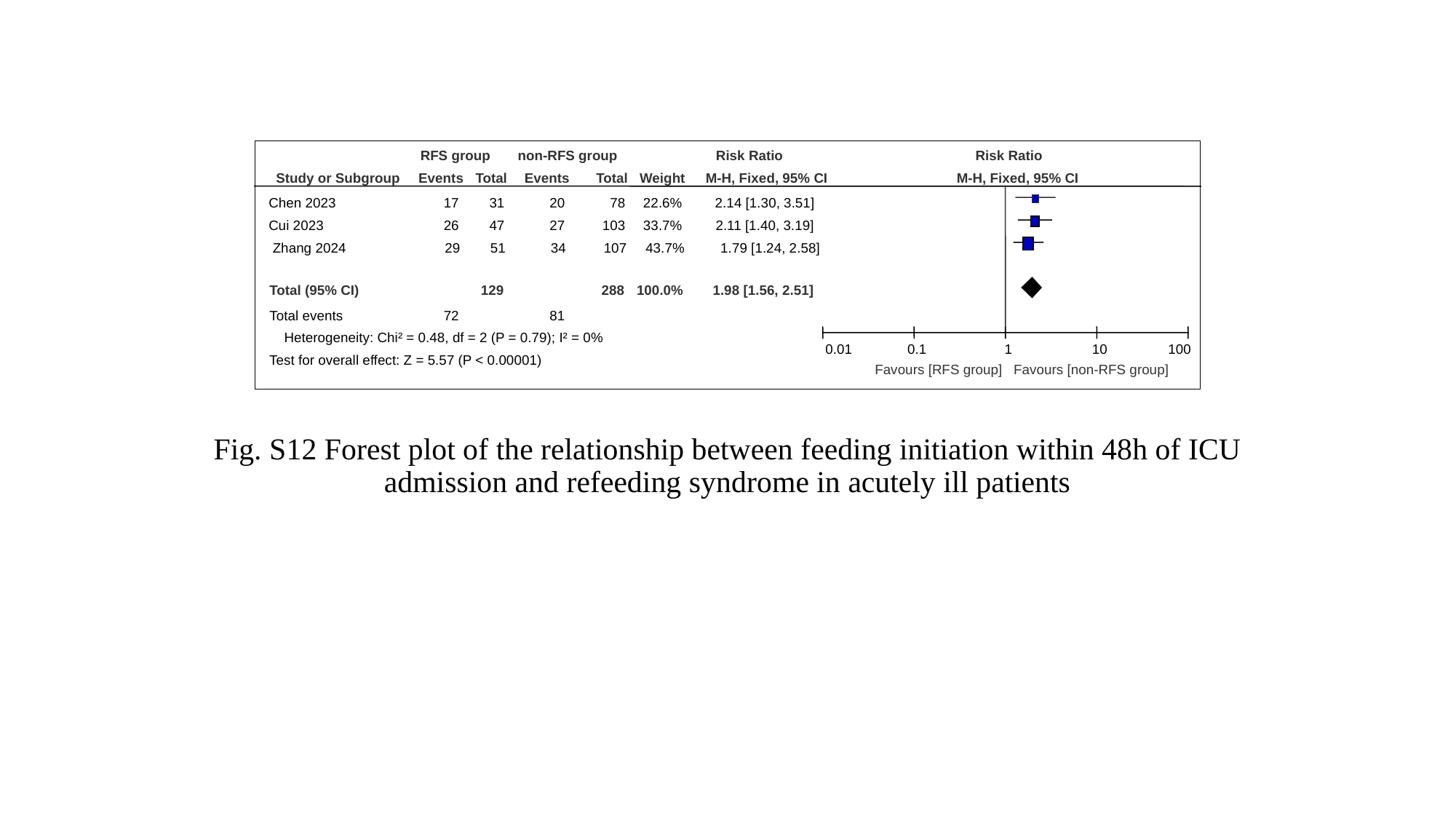

RFS group
non-RFS group
Risk Ratio
Risk Ratio
Study or Subgroup
Events
Total
Events
Total
Weight
M-H, Fixed, 95% CI
M-H, Fixed, 95% CI
Chen 2023
17
31
20
78
22.6%
2.14 [1.30, 3.51]
Cui 2023
26
47
27
103
33.7%
2.11 [1.40, 3.19]
Zhang 2024
29
51
34
107
43.7%
1.79 [1.24, 2.58]
Total (95% CI)
129
288
100.0%
1.98 [1.56, 2.51]
Total events
72
81
Heterogeneity: Chi² = 0.48, df = 2 (P = 0.79); I² = 0%
0.01
0.1
1
10
100
Test for overall effect: Z = 5.57 (P < 0.00001)
Favours [RFS group]
Favours [non-RFS group]
Fig. S12 Forest plot of the relationship between feeding initiation within 48h of ICU admission and refeeding syndrome in acutely ill patients
