# Supplementary material for: Meta‐Analysis of Refeeding Syndrome in Predicting the Risk of Occurrence in Critically Ill Patients
Source: J Nutr Metab. 2026 Feb 18;2026:6660254. doi: 10.1155/jnme/6660254 (PMC12917335; doi:10.1155/jnme/6660254)
Supplement: Supplementary file 13 — Supporting Information 13 Figure S13: Forest plot of the relationship between history of diabetes and refeeding syndrome in acutely ill patients. Three studies [14, 16, 19] reported a history of diabetes, and the meta‐analysis showed heterogeneity among the studies (I 2 = 89%, p < 0.01). Therefore, the analysis was carried out using the random‐effects model, and the results showed that the difference was statistically significant [OR = 2.53, 95% CI (1.18, 5.44), p = 0.02], suggesting that a history of diabetes can be a risk factor for predicting the development of refeeding syndrome in acutely ill patients. [file JNME-2026-6660254-s006.pptx]

## Slide 1
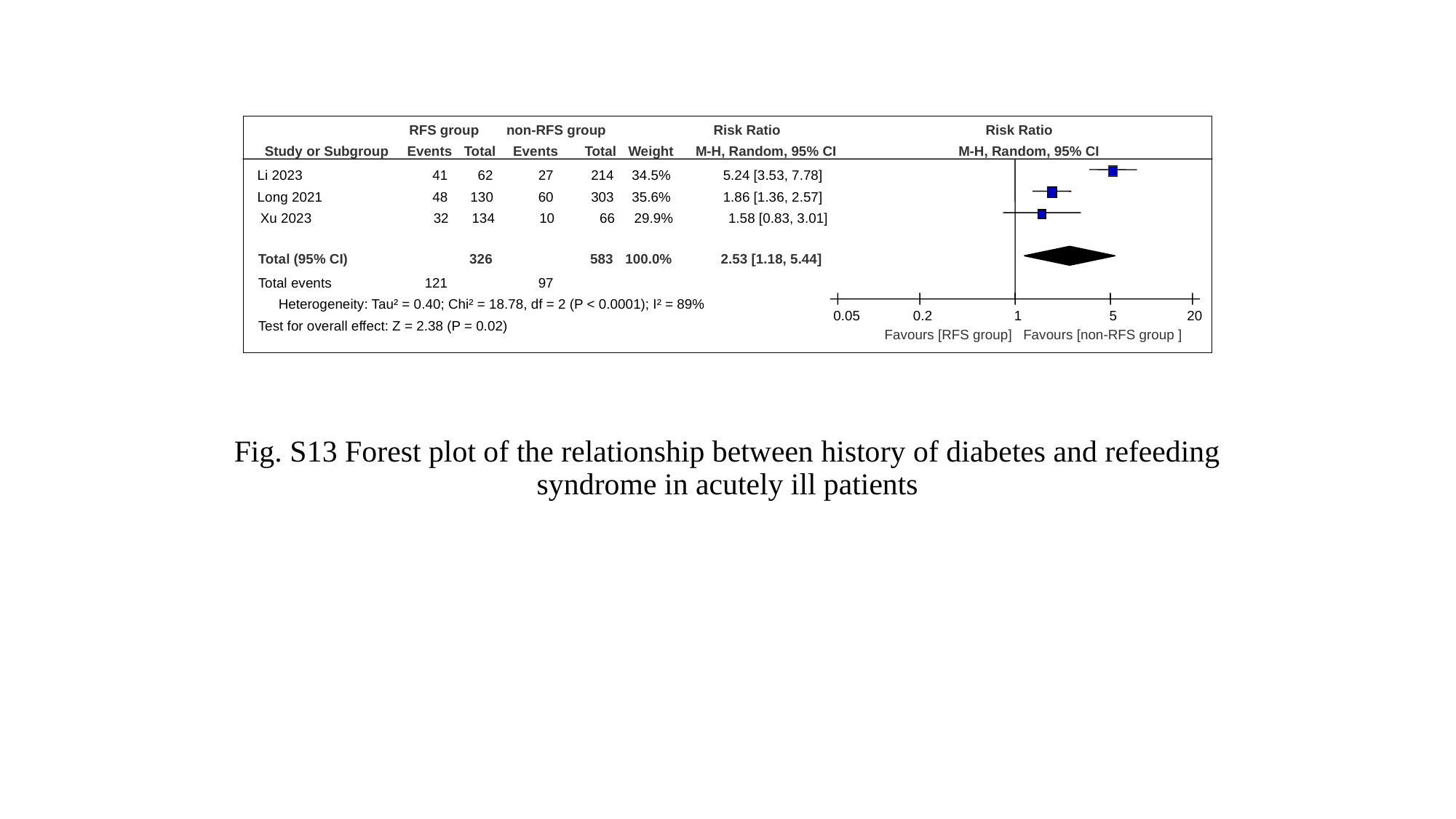

RFS group
non-RFS group
Risk Ratio
Risk Ratio
Study or Subgroup
Events
Total
Events
Total
Weight
M-H, Random, 95% CI
M-H, Random, 95% CI
Li 2023
41
62
27
214
34.5%
5.24 [3.53, 7.78]
Long 2021
48
130
60
303
35.6%
1.86 [1.36, 2.57]
Xu 2023
32
134
10
66
29.9%
1.58 [0.83, 3.01]
Total (95% CI)
326
583
100.0%
2.53 [1.18, 5.44]
Total events
121
97
Heterogeneity: Tau² = 0.40; Chi² = 18.78, df = 2 (P < 0.0001); I² = 89%
0.05
0.2
1
5
20
Test for overall effect: Z = 2.38 (P = 0.02)
Favours [RFS group]
Favours [non-RFS group ]
Fig. S13 Forest plot of the relationship between history of diabetes and refeeding syndrome in acutely ill patients
