# Supplementary material for: Meta‐Analysis of Refeeding Syndrome in Predicting the Risk of Occurrence in Critically Ill Patients
Source: J Nutr Metab. 2026 Feb 18;2026:6660254. doi: 10.1155/jnme/6660254 (PMC12917335; doi:10.1155/jnme/6660254)
Supplement: Supplementary file 14 — Supporting Information 14 Table S1: Comparison table of risk factors in this study and NICE/ASPEN guidelines. This table is designed to compare the consistency between the RFS‐related risk factors identified in this study and those specified in two international authoritative guidelines. The table details the consistency level of each risk factor with the respective guidelines, supplemented by key notes clarifying the clinical significance of the factors and differences in guideline focus. [file JNME-2026-6660254-s007.docx]

| **Table S1** Comparison table of risk factors in this study and NICE/ASPEN guidelines | | | |
| --- | --- | --- | --- |
| Risk Factors Identified in This Study | Consistency with NICE Guidelines | Consistency with ASPEN Guidelines | Notes |
| Low serum phosphorus level | Not mentioned  (NICE focuses on pre-feeding depletion, not post-feeding electrolyte changes) | Highly consistent  (ASPEN lists hypophosphatemia as a core metabolic marker of RFS) | Key indicator reflecting metabolic disturbance during refeeding |
| Low serum albumin level | Partially consistent  (albumin is a nutritional marker, but NICE prioritizes weight/BMI) | Highly consistent  (reflects disease severity and systemic inflammation) | In ICU patients, it is more a marker of disease severity than pure nutritional depletion |
| Inappropriate daily protein/calorie intake | Low consistency  (NICE focuses on pre-feeding depletion, not feeding process supply) | Highly consistent  (core of dynamic nutritional delivery assessment in ASPEN) | Reflects the tolerance of metabolic system to nutritional supply |
| Elevated APACHE II score | No consistency  (NICE does not involve disease severity scores) | Highly consistent  (direct indicator of disease severity emphasized by ASPEN) | Reflects the overall severity of critically ill patients |
| Elevated SOFA score | No consistency  (NICE does not involve organ function assessment) | Highly consistent  (ASPEN emphasizes organ function status in RFS assessment) | Related to metabolic capacity of organ system |
| Advanced age | Not mentioned | Not explicitly emphasized (supplementary risk factor) | Related to age-related metabolic decline |
| Initiation of feeding within 48h of ICU admission | No consistency  (NICE does not specify feeding timing) | Highly consistent  (ASPEN focuses on dynamic adjustment of feeding timing) | Reflects the rationality of early nutritional intervention timing |
| History of diabetes | Not mentioned | Not explicitly emphasized (supplementary risk factor) | Related to pre-existing metabolic disorders |
