# Supplementary material for: Meta‐Analysis of Refeeding Syndrome in Predicting the Risk of Occurrence in Critically Ill Patients
Source: J Nutr Metab. 2026 Feb 18;2026:6660254. doi: 10.1155/jnme/6660254 (PMC12917335; doi:10.1155/jnme/6660254)
Supplement: Supplementary file 15 — Supporting Information 15 Table S2: Subgroup analysis. Subgroup analyses of the association between refeeding syndrome (RFS) development and serum phosphorus, albumin, prealbumin levels, and APACHE II scores in critically ill patients. [file JNME-2026-6660254-s008.docx]

| Subgroup analysis of the relationship between the development of refeeding syndrome and baseline serum phosphate in critically ill patients | | | | | | | | | | |
| --- | --- | --- | --- | --- | --- | --- | --- | --- | --- | --- |
|  | Subgroup | n | Heterogeneity | | Model selection | Meta-analysis results | | | Test for subgroup difference | |
|  |  |  | I^2^(%) | P |  | MD | 95%CI | P | I^2^(%) | P |
| Study design | retrospective study | 2 | 0 | 0.70 | fixed | -0.22 | -0.28~-0.16 | <0.001 | 97.4 | <0.001 |
|  | prospective study | 3 | 0 | 0.78 | fixed | -0.01 | -0.03~0.01 | 0.34 |  |  |
| Definitions of RFS | the change in electrolyte levels + clinical symptoms | 2 | 97 | <0.001 | Random | -0.11 | -0.30~0.09 | 0.28 | 0 | 0.95 |
|  | the change in electrolyte levels | 3 | 79 | 0.009 | Random | -0.10 | -0.25~0.05 | 0.18 |  |  |
| Mean age | >65 | 2 | 0 | 0.70 | fixed | -0.22 | -0.28~-0.16 | <0.001 | 97.4 | <0.001 |
|  | <65 | 3 | 0 | 0.78 | fixed | -0.01 | -0.03~0.01 | 0.34 |  |  |
| Nutritional Mode | EN | 3 | 89 | <0.001 | Random | -0.10 | -0.25~0.05 | 0.20 | 0 | 0.91 |
|  | EN+PN | 2 | 91 | 0.001 | Random | -0.12 | -0.34~0.11 | 0.31 |  |  |

| Subgroup analysis of the relationship between the development of refeeding syndrome and baseline serum albumin in critically ill patients | | | | | | | | | | |
| --- | --- | --- | --- | --- | --- | --- | --- | --- | --- | --- |
|  | Subgroup | n | Heterogeneity | | Model selection | Meta-analysis results | | | Test for subgroup difference | |
|  |  |  | I^2^(%) | P |  | MD | 95%CI | P | I^2^(%) | P |
| Study design | retrospective study | 3 | 93 | <0.001 | Random | -4.31 | -7.48~-1.14 | 0.008 | 85 | 0.01 |
|  | Prospective study | 4 | 1 | 0.39 | fixed | -0.09 | -0.55~0.37 | 0.70 |  |  |
| Study method | case-control study | 3 | 93 | <0.001 | Random | -4.31 | -7.48~-1.14 | 0.008 | 85 | 0.01 |
|  | cohort study | 4 | 1 | 0.39 | fixed | -0.09 | -0.55~0.37 | 0.70 |  |  |
| Definitions of RFS | the change in electrolyte levels + clinical symptoms | 3 | 97 | <0.001 | Random | -3.85 | -8.26~0.57 | 0.09 | 43.9 | 0.18 |
|  | the change in electrolyte levels | 4 | 44 | 0.15 | fixed | -0.59 | -1.26~0.09 | 0.09 |  |  |
| Mean age | >65 | 2 | 0 | <0.001 | fixed | -1.18 | -1.43~-0.92 | <0.001 | 98.4 | <0.001 |
|  | <65 | 4 | 0 | 0.56 | fixed | -0.03 | -0.15~0.08 | 0.56 |  |  |
| Nutritional Mode | EN | 4 | 90 | <0.001 | Random | -1.95 | -4.08~0.19 | 0.07 | 0 | 0.85 |
|  | EN+PN | 3 | 96 | <0.001 | Random | -2.43 | -7.03~2.17 | 0.30 |  |  |
| Country | China | 5 | 95 | <0.001 | Random | -2.35 | -4.71~0.01 | 0.05 | 0 | 0.53 |
|  | non-China | 2 | 0 | 0.005 | fixed | -1.52 | -2.58~-0.47 | 0.005 |  |  |

| Subgroup analysis of the relationship between the development of refeeding syndrome and baseline serum prealbumin level in critically ill patients | | | | | | | | | | |
| --- | --- | --- | --- | --- | --- | --- | --- | --- | --- | --- |
|  | Subgroup | n | Heterogeneity | | Model selection | Meta-analysis results | | | Test for subgroup difference | |
|  |  |  | I^2^(%) | P |  | MD | 95%CI | P | I^2^(%) | P |
| Study design | retrospective study | 2 | 97 | <0.001 | Random | -53.06 | -119.74~13.62 | 0.12 | 62.7 | 0.1 |
|  | Prospective study | 3 | 49 | 0.14 | fixed | 1.90 | -1.78~5.58 | 0.31 |  |  |
| Study method | case-control study | 2 | 97 | <0.001 | Random | -53.06 | -119.74~13.62 | 0.12 | 62.7 | 0.1 |
|  | cohort study | 3 | 49 | 0.14 | fixed | 1.90 | -1.78~5.58 | 0.31 |  |  |
| Definitions of RFS | the change in electrolyte levels + clinical symptoms | 3 | 98 | <0.001 | Random | -32.30 | -59.14~-5.45 | 0.02 | 82.6 | 0.02 |
|  | the change in electrolyte levels | 2 | 74 | 0.05 | Random | 9.08 | -11.56~29.71 | 0.39 |  |  |
| Mean age | >65 | 2 | 97 | <0.001 | Random | -53.06 | -119.74~13.62 | 0.12 | 62.7 | 0.1 |
|  | <65 | 3 | 49 | 0.14 | fixed | 1.90 | -1.78~5.58 | 0.31 |  |  |
| Nutritional Mode | EN | 2 | 95 | <0.001 | Random | -9.74 | -29.70~10.22 | 0.34 | 0 | 0.85 |
|  | EN+PN | 3 | 97 | <0.001 | Random | -21.02 | -73.97~31.92 | 0.4 |  |  |

| Subgroup analysis of the relationship between the development of refeeding syndrome and APACHE II score in critically ill patients | | | | | | | | | | |
| --- | --- | --- | --- | --- | --- | --- | --- | --- | --- | --- |
|  | Subgroup | n | Heterogeneity | | Model selection | Meta-analysis results | | | Test for subgroup difference | |
|  |  |  | I^2^(%) | P |  | MD | 95%CI | P | I^2^(%) | P |
| Study method | case-control study | 4 | 71 | 0.02 | Random | 3.38 | 2.17~4.59 | 0.02 | 85.1 | 0.01 |
|  | cohort study | 2 | 60 | 0.12 | Random | 0.99 | -0.34~2.33 | 0.12 |  |  |
| Definitions of RFS | the change in electrolyte levels + clinical symptoms | 3 | 95 | <0.001 | Random | 3.07 | 0.31~5.84 | 0.03 | 0 | 0.65 |
|  | the change in electrolyte levels | 3 | 27 | 0.25 | fixed | 2.45 | 1.67~3.23 | <0.001 |  |  |
| Mean age | >65 | 3 | 56 | 0.11 | Random | 2.50 | 1.35~3.64 | <0.001 | 0 | 0.85 |
|  | <65 | 2 | 90 | <0.001 | Random | 2.99 | -2.08~8.06 | 0.25 |  |  |
| Nutritional Mode | EN | 2 | 0 | 0.75 | Random | 3.30 | 2.56~4.05 | <0.001 | 0 | 0.37 |
|  | EN+PN | 4 | 89 | <0.001 | fixed | 2.33 | 0.37~4.30 | 0.02 |  |  |
| Country | China | 5 | 92 | <0.001 | Random | 2.73 | 0.67~4.79 | 0.009 | 0 | 0.96 |
|  | non-China | 2 | 35 | 0.21 | fixed | 2.74 | 1.81~3.68 | <0.001 |  |  |

**Table S2 Sub-group analysis**
