# Supplementary material for: Meta‐Analysis of Refeeding Syndrome in Predicting the Risk of Occurrence in Critically Ill Patients
Source: J Nutr Metab. 2026 Feb 18;2026:6660254. doi: 10.1155/jnme/6660254 (PMC12917335; doi:10.1155/jnme/6660254)
Supplement: Supplementary file 16 — Supporting Information 16 Table S3: Leave‐one‐out sensitivity analysis. This table presents the results of leave‐one‐out sensitivity analysis examining the robustness of our meta‐analysis findings. The analysis was performed by systematically excluding each included study one at a time to evaluate their individual influence on the pooled effect estimates. [file JNME-2026-6660254-s011.docx]

| Table S2 Leave-one-out sensitivity analysis | | | |
| --- | --- | --- | --- |
| Predictive risk factors | Original | Deletion of the study | present |
| Serum phosphorus levels | 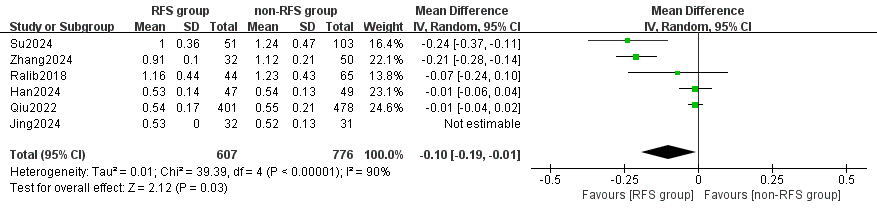 | Zhang et al^[8]^ | 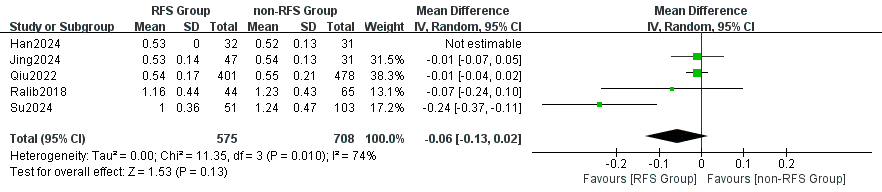 |
| Serum albumin level | 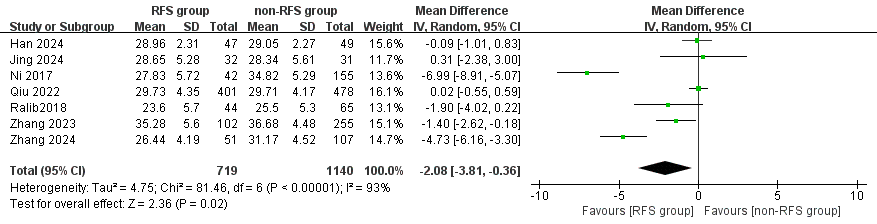 | Ni et al^[17]^ | 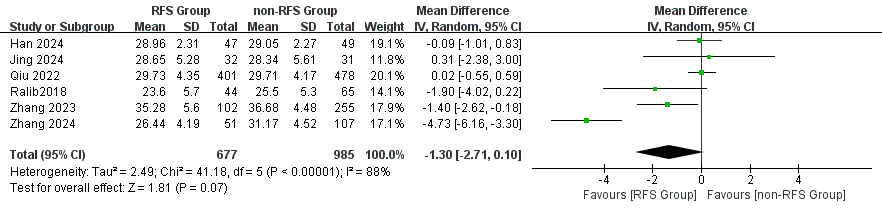 |
| APACHE II score | 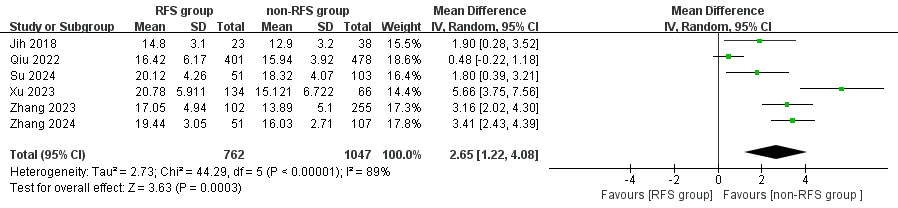 | Qiu et al^[20]^ | 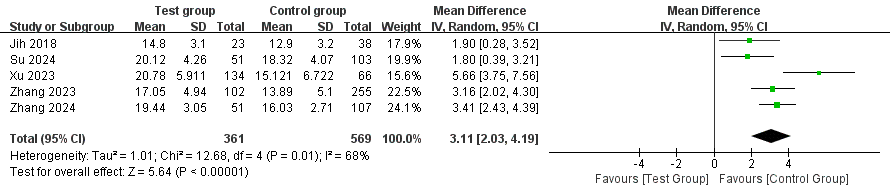 |
| History of diabetes | 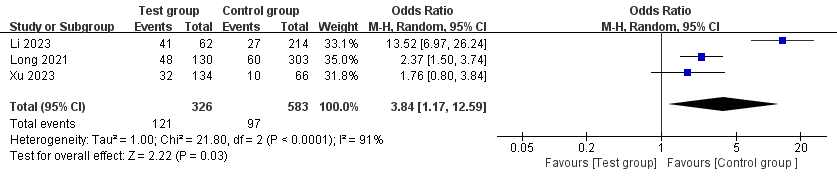 | Li et al^[14]^ | 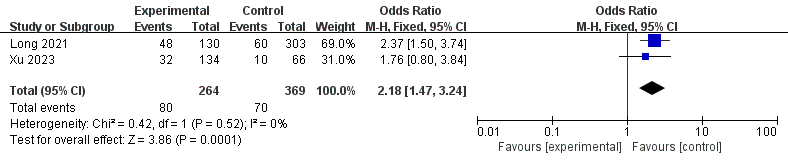 |
| Serum magnesium levels | Robust results | | |
| Serum potassium levels | Robust results | | |
| Serum prealbumin level | Robust results | | |
| Daily protein intake | Robust results | | |
| Daily calorie intake | Robust results | | |
| NRS2002 score | Robust results | | |
| SOFA score | Robust results | | |
| Age | Robust results | | |
| Feeding started within 48 h of admission to ICU | Robust results | | |
